# Validation of the Hungarian FAD-GF and BAFFS in a Multi-Informant Clinical Sample of Families of Adolescents

**DOI:** 10.3390/bs16101753

**Published:** 2026-09-25

**Authors:** Noémi Gyenis-Kátay, Ildikó Danis, Klára Soltész-Várhelyi, András Szauer, Szabolcs Török

**Affiliations:** 1 Interdisciplinary Social Sciences Program, Mental Health Sciences Division, Doctoral College of Semmelweis University, 1089 Budapest, Hungary; gyenis-katay.noemi@bethesda.hu (N.G.-K.); danis.ildiko@semmelweis.hu (I.D.); szauer.andras@bethesda.hu (A.S.); 2 Early Intervention and Diagnostic Service (KIDSz), Bethesda Children’s Hospital of the Reformed Church in Hungary, 1146 Budapest, Hungary; 3 Faculty of Health and Public Services, Institute of Mental Health, Semmelweis University, 1089 Budapest, Hungary; 4 Faculty of Humanities and Social Sciences, Institute of Psychology, Pázmány Péter Catholic University, 1088 Budapest, Hungary; soltesz-varhelyi.klara@btk.ppke.hu

**Keywords:** validation, clinical sample, Family Assessment Device, BAFFS, multi-informant assessment

## Abstract

Family functioning is central to youth adaptation, yet brief measures require validation across informants and clinical contexts. This study evaluated the Hungarian 12-item Family Assessment Device–General Functioning Scale (FAD-GF) and the three-item Brief Assessment of Family Functioning Scale (BAFFS) in 562 respondents from 213 families of adolescents with autism spectrum disorder (ASD), anorexia nervosa (AN), or diabetes mellitus (DM). Analyses included cluster-adjusted ordinal confirmatory and multigroup CFA, exploratory factor and general-factor analyses, internal-consistency estimation, and cluster-robust external-association tests. Prespecified FAD-GF models showed inadequate fit, but exploratory analyses indicated a dominant general factor accompanied by recurring local dependencies. An exploratory one-factor model with seven simultaneously estimated residual covariances showed broadly acceptable fit (CFI = 0.962, TLI = 0.946, RMSEA = 0.078, SRMR = 0.034). Internal consistency was high for the FAD-GF (α = 0.893, ωT = 0.896) and acceptable for the BAFFS (α = 0.747, ωT = 0.756). The FAD-GF supported partial scalar invariance across parents and adolescents, strict invariance across fathers and mothers, and scalar but not strict invariance across ASD and AN parents. The BAFFS supported scalar but not strict invariance across fathers and mothers and metric but not scalar invariance across ASD and AN parents; adolescent-group solutions were inadmissible, and its just-identified CFA could not test unidimensionality. Both scales showed expected associations with stress, well-being, relationship satisfaction, and coping. The findings support use of the FAD-GF total score, whereas the BAFFS is better retained as a brief observed index, particularly in parental reports, pending further cross-group validation.

## 1. Introduction

Family functioning can be understood in terms of recurring patterns of communication, emotional climate, problem solving, role organization, rules, and mutual responsiveness within the family system. These patterns are associated with children’s and adolescents’ psychological well-being, health-related behaviors, and adaptation in the context of chronic or psychological conditions (Thomas et al., 2017; Yap et al., 2014; Berge et al., 2014; Izzo et al., 2022). Difficulties in communication, emotional support, and relational functioning may therefore be clinically relevant across a range of pediatric contexts. Brief and reliable assessment of family functioning is consequently important in child and adolescent clinical practice, health psychology, and family therapy.

Several self-report instruments have been developed to assess family functioning, including the Family Functioning Index (Pless & Satterwhite, 1973), the Family APGAR (Smilkstein, 1978), the Family Assessment Measure (Skinner et al., 1983), the Family Environment Scale (Moos et al., 1974), the Family Adaptability and Cohesion Evaluation Scales (FACES; Olson et al., 1978), and the Family Assessment Device (FAD; Epstein et al., 1983). The FAD is one of the most widely used measures of family functioning and is based on the McMaster Model of Family Functioning (Epstein et al., 1978; Epstein et al., 1983; Hamilton & Carr, 2016). The model describes six dimensions of family functioning: problem solving, communication, roles, affective responsiveness, affective involvement, and behavior control. These domains address how families resolve difficulties, exchange information, organize responsibilities, respond emotionally to one another, remain involved in one another’s lives, and regulate behavior through rules and expectations. The full FAD contains 60 items and includes the 12-item General Functioning scale (FAD-GF), which provides a global assessment of family functioning across these domains. The FAD-GF comprises six positively worded and six negatively worded items.

The FAD-GF was developed as a brief global measure of family functioning that summarizes information across the broader domains represented in the full FAD (Epstein et al., 1983; Miller et al., 1985). Early validation studies reported good internal consistency and supported its ability to distinguish between clinical and non-clinical family samples (Miller et al., 1985; Byles et al., 1988). Subsequent work has supported its continued use in both community and clinical research (Mansfield et al., 2015). Because it can be administered independently of the full 60-item FAD, the 12-item scale has been particularly useful when a global estimate of family functioning is required without detailed assessment of each McMaster domain (Byles et al., 1988). Its psychometric properties have since been examined across a wide range of linguistic and cultural contexts, including Chinese, Swedish, Portuguese, Norwegian, Malay, Indonesian, Turkish, Arabic and African samples (Shek, 2001; Bylund et al., 2016; Pires et al., 2016; Hausken et al., 2019; Almeida et al., 2020; Babar et al., 2021; Setiana & Tondok, 2024; Körün et al., 2025; Assaf et al., 2025; Alaofè et al., 2026).

Evidence from different populations indicates that the internal structure of the FAD-GF is not entirely consistent across samples and cultural contexts (Boterhoven de Haan et al., 2015; Badovinac et al., 2024; Assaf et al., 2025). Although the 12-item one-factor model remains the most widely used representation, several studies have supported alternative item selections or multidimensional solutions. The six-item positively worded FAD-GF6+ showed favorable psychometric properties in a large Australian population sample (Boterhoven de Haan et al., 2015), and Assaf et al. (2025) identified this version as the most suitable among several previously proposed models in an Arabic-speaking Lebanese sample. Babar et al. (2021) reported a two-factor solution separating positively and negatively worded items in a Malay adaptation, while Badovinac et al. (2024) found support for more than one plausible representation of the scale in a clinic-referred sample. In an Indonesian adolescent sample, Setiana and Tondok (2024) proposed a nine-item version after identifying weak item-total associations for several negatively worded items. This variability suggests that the dimensionality of the FAD-GF, the functioning of individual items, and the performance of shortened versions should be evaluated empirically in each new clinical and cultural context.

Among the shortened forms of the FAD-GF, the Brief Assessment of Family Functioning Scale (BAFFS) is an ultra-brief three-item measure developed by Mansfield et al. (2019). It comprises three items drawn from the FAD-GF, including two positively worded items and one negatively worded item. The scale was developed to provide a concise indicator of global family functioning when respondent burden needs to be minimized. In the original validation study, the BAFFS showed acceptable internal consistency and strong associations with the 12-item FAD-GF and with external variables (Mansfield et al., 2019). A subsequent German validation provided additional support for its psychometric performance in a population-based sample (Spitzer et al., 2022). Compared with the FAD-GF and other shortened forms, however, independent evidence on the BAFFS remains relatively limited, particularly across clinical and multi-informant contexts. Further evaluation of its structure, reliability, and cross-group comparability is therefore warranted in new populations and linguistic settings.

Multi-informant assessment is particularly relevant when family functioning is evaluated in child and adolescent clinical contexts. Reports from different informants may diverge because family members observe and experience the same relational system from different developmental and role-based perspectives (De Los Reyes & Kazdin, 2005; De Los Reyes, 2011; De Los Reyes et al., 2013). Previous FAD studies have similarly documented both shared and informant-specific components in reports from parents and adolescents, as well as differences between maternal and paternal ratings (Sawyer et al., 1988; Georgiades et al., 2008; Bagley et al., 2001). Children’s and adolescents’ reports may therefore provide information that is not fully redundant with parental reports, although the reliability of some FAD dimensions may be weaker among younger respondents (Bihun et al., 2002). When scores are compared across family roles, however, observed differences are interpretable only to the extent that the instrument measures the underlying construct comparably across groups. Measurement invariance testing is therefore needed to distinguish substantive differences in reported family functioning from differences arising from the measurement properties of the scale (Chen, 2007; Vandenberg & Lance, 2000).

To date, no Hungarian clinical study has jointly examined the internal structure, reliability, external associations, and measurement invariance of the FAD-GF and BAFFS in a multi-informant clinical sample of families of adolescents. Evaluating these properties across different clinical contexts is important because the interpretation and comparability of family-functioning scores cannot be assumed to generalize automatically across populations. The present study therefore included families of adolescents with autism spectrum disorder (ASD), anorexia nervosa (AN), and diabetes mellitus (DM), representing distinct clinical contexts in which family functioning is relevant to assessment and care.

Previous research has linked poorer family functioning with higher perceived stress, lower psychological well-being, and less favorable relationship satisfaction and stress-management experiences (Thomas et al., 2017; Staccini et al., 2014). These constructs were therefore included as theoretically expected external correlates of perceived family functioning. Examining these associations can provide complementary information about how FAD-GF and BAFFS scores relate to broader aspects of psychological and relational adjustment in the present clinical sample.

Because the present sample included multiple respondent roles and distinct clinical groups, it was important to examine whether FAD-GF and BAFFS scores could be interpreted comparably across these groups. Psychometric findings obtained in one linguistic, clinical, or respondent context cannot be assumed to generalize automatically to another, particularly for shortened or adapted measures (Furr, 2011).

The aim of the present study was to examine the psychometric properties of the Hungarian FAD-GF and BAFFS in a multi-informant clinical sample of families of adolescents. For the FAD-GF, we evaluated several previously proposed measurement models and, where these proved inadequate, further examined dimensionality, general-factor strength, and local item dependencies. We also assessed the internal consistency of both measures and examined their associations with perceived stress, psychological well-being, relationship satisfaction, and stress-management indicators as theoretically expected external correlates. Measurement invariance was evaluated across parents and adolescents, fathers and mothers, and index adolescents and siblings. Diagnostic-group invariance was examined among parents in the ASD and AN groups; the smaller DM parental sample was retained for descriptive, reliability, and external-association analyses but was not included in diagnostic multigroup CFA. We expected the FAD-GF to show adequate internal consistency and a substantial general family-functioning component, while allowing for the possibility of localized item dependencies. Given the more limited independent evidence available for the BAFFS, its structure and cross-group performance were evaluated more cautiously.

## 2. Materials and Methods

### 2.1. Research Design and Sampling Procedures

This study uses data from a larger, nationwide clinical initiative (the “Gyermekgyógyító Plusz”/“Child-Healing Plus” program), funded by the MOL Foundation. This large-scale project provides systemic family therapy across various pediatric conditions, including neurodevelopmental, behavioral, emotional, and eating disorders, as well as childhood malignancies and chronic somatic illnesses. The initiative established an extensive network of family therapists within public healthcare and specialized social institutions across Hungary. At the time of analysis, the program included 20 participating therapeutic centers nationwide, encompassing major university clinics, national pediatric institutes, and specialized foundations. Data collection is continuous; the current sample includes data collected between April 2023 and March 2026.

For the current psychometric analysis, we selected three diagnostically homogeneous subgroups. Specifically, the analysis focuses on families where the index patient was an adolescent with a pre-existing, formal diagnosis of autism spectrum disorder, anorexia nervosa, or diabetes mellitus. These diagnoses were established prior to the study by clinical specialists (pediatricians, psychiatrists, or clinical psychologists) according to international diagnostic standards (e.g., DSM or ICD).

Questionnaires were administered to parents (fathers and mothers) and their adolescent children aged 12 and older (both index patients and siblings). To ensure the validity and homogeneity of adult–adolescent invariance testing, individuals in a child role (e.g., older siblings) over 18 years of age (n = 12) were excluded from the current analysis. This exclusion maintained a consistent developmental comparison between generational groups, ensuring that the data obtained from the children strictly represented the adolescent population. Additionally, because questionnaire completion was entirely voluntary and not a prerequisite for therapy, data were not obtained from all family members within the participating households.

Recruitment was conducted locally by clinicians at the participating treatment centers, who invited families entering the family therapy intervention. However, because the research team received only the resulting study dataset and no center-level screening or recruitment records that could be linked to individual study entries, the numbers of eligible, approached, or declining families and family members could not be reconstructed, and a recruitment response rate could not be calculated.

Data collection followed a standardized, multi-wave protocol across the participating institutions. Families were assigned unique ID codes to ensure anonymity. Questionnaire packages were administered at three specific intervals: at baseline (pre-treatment), after the seventh session, and at termination. The present analysis focuses exclusively on the baseline measurement.

Standardized data collection took place at the clinical sites. Participants completed assessments either via a secure online survey link provided by therapists or via paper-and-pencil format. Family members were requested to complete questionnaires simultaneously but independently to avoid mutual influence. Paper responses were entered into the same electronic system by staff at the participating centers. Because the original administration mode was not retained at the respondent level in the dataset supplied to the research team, mode effects could not be examined.

The study followed the Declaration of Helsinki and received ethical approval from the Medical Research Council’s Scientific and Research Ethics Committee (ETT TUKEB; case number BM/6095-4/2023). Participation was strictly voluntary, and participants could withdraw at any time without consequence to their therapeutic care. Written informed consent was obtained from all adult participants and legal guardians, while adolescents provided written assent.

### 2.2. Participants

The total sample comprised 562 respondents from 213 Hungarian families. Families were recruited across three diagnostic groups defined by the index adolescent’s condition: autism spectrum disorder (ASD; 78 families), anorexia nervosa (AN; 98 families), and diabetes mellitus (DM; 37 families). The sample included 159 fathers, 207 mothers, 126 index adolescents, and 70 siblings. Participant flow and sample composition are summarized in Figure 1, while a detailed cross-tabulation of respondent configurations within families is provided in Supplementary Materials A (Table S1). Detailed demographic characteristics disaggregated by clinical group and family role are presented in Table 1.

The diagnostic subgroups exhibited distinct demographic profiles. The ASD group (77 mothers, 69 fathers, and 22 adolescents) included relatively younger parents, with approximately 60% having completed higher education, and a balanced gender distribution among adolescents. The AN group (82 fathers, 95 mothers, 142 adolescents) provided the largest adolescent subsample, characterized by a strong female predominance and the highest average parental age. The DM group (8 fathers, 35 mothers, and 32 adolescents) was the smallest and, unlike the other two groups, consisted entirely of families residing outside the capital (predominantly in regional cities and towns). The gender ratio among adolescents was 40.6% male and 59.4% female.

The average number of children per family in the total sample was 2.14 (*SD* = 0.85, *Max* = 5). Notably, the number of children significantly differed by diagnostic group, *F*(2, 208) = 9.54, *p* < 0.001, *η*^2^ = 0.08. Families in the DM group had significantly higher average number of children (*M* = 2.54, *SD* = 0.84) than the AN group (*M* = 2.21, *SD* = 0.79, *p* = 0.008) or the ASD group (*M* = 1.87, *SD* = 0.84, *p* < 0.001).

### 2.3. Measures

#### 2.3.1. Family Functioning: General Functioning Subscale of the Family Assessment Device (FAD-GF) and Brief Assessment of Family Functioning Scale (BAFFS)

Perceived family functioning was evaluated using the Hungarian version of the FAD (Epstein et al., 1983). The original 60-item instrument contains six specific dimensions and a global scale. This study focuses on the 12-item FAD-GF (comprising items 1, 6, 11, 16, 21, 26, 31, 36, 41, 46, 51, and 56), which provides a global assessment of family functioning. Additionally, we evaluated the BAFFS (Mansfield et al., 2019), an ultra-brief 3-item measure derived from the FAD-GF (comprising items 26, 51, and 56) and developed to provide a concise indicator of global family functioning while minimizing respondent burden. All items are rated on a 4-point Likert scale ranging from 1 (strongly agree) to 4 (strongly disagree). After reverse-scoring the negatively worded items, higher mean scores indicate greater perceived family dysfunction. The Hungarian wording used in the present study and the detailed scoring instructions for the FAD-GF and BAFFS are available at the following link, with the permission of G. I. Keitner: https://semmelweis.hu/mental/files/2026/09/FAD_GF_BAFFS_magyar-forditas_instrukcio_kozzeteve_2026.pdf, (accessed on 1 September 2026). The document also provides the revised Hungarian wording of FAD1, agreed upon following the present analyses and recommended for future use. The data analyzed in this study were collected using the earlier Hungarian wording of FAD1.

The original FAD development study provided initial psychometric support for the full instrument, including the General Functioning scale (Epstein et al., 1983). Subsequent studies provided further evidence for the reliability, validity, and discriminative performance of the FAD-GF (Byles et al., 1988; Miller et al., 1985). The original BAFFS validation reported acceptable internal consistency for the three-item scale (α = 0.72; Mansfield et al., 2019). The Hungarian translations and first adaptations of the 60-item FAD were conducted in several waves from the late 1980s. In accordance with international guidelines at the time, the process involved two independent translations, a consensus review, a back-translation and a verification. The final version was approved by G. Keitner from the FAD’s American developers. Pilot studies were also conducted. The initial version was utilized in American–Hungarian comparative studies (Keitner et al., 1990, 1991) and a Hungarian PhD study (Kurimay, 1994). Later, another Hungarian research team (J. Gervai and colleagues) used the FAD in a study involving parents of young children in the late 1990s and early 2000s. At that time, the text underwent further refinement and was approved by G. Keitner, again. Basic psychometric data were also published in Hungarian (Danis et al., 2005). The finalized Hungarian version—in cooperation with the 3 Hungarian groups—was later used in a large nationally representative community study. The preliminary psychometric findings were presented by Danis et al. (2022); the publication on the psychometric properties and validation is currently in preparation.

In the present article, we only used the 12-item FAD-GF and the 3-item BAFFS. While their preliminary psychometric findings were based on a representative non-clinical sample, this study extends the validation by testing the performance and multigroup invariance of both the 12-item FAD-GF and 3-item BAFFS across distinct clinical groups and generational roles.

#### 2.3.2. WHO-5 Well-Being Index: Hungarian WBI-5 Adaptation

Subjective psychological well-being was assessed using the Hungarian adaptation of the five-item WHO Well-Being Index (WBI-5; Susánszky et al., 2006), a shortened version of the WHO Well-Being Index described by Bech et al. (1996). This unidimensional self-report measure assesses positive psychological well-being during the previous two weeks. In the Hungarian adaptation used in the present study, items are rated on a four-point scale ranging from 0 (not at all characteristic) to 3 (completely characteristic), yielding a total score ranging from 0 to 15. Higher scores indicate greater psychological well-being. The scale showed acceptable internal consistency in the present sample (Cronbach’s α = 0.78).

#### 2.3.3. Perceived Stress Scale (PSS-4)

To quantify perceived stress, the 4-item short form of the PSS-4 (Cohen & Williamson, 1988) was employed, based on the Hungarian validation by Stauder and Konkolÿ Thege (2006). The instrument assesses the degree to which respondents find their recent life circumstances to be unmanageable, unpredictable, or taxing. Items are scored on a 5-point Likert scale; following the reverse-coding of appropriate items, a higher score signifies a greater perception of stress. The internal consistency within the present sample was found to be good (α = 0.78).

#### 2.3.4. Relationship Satisfaction and Dyadic Coping

To examine expected external associations of the FAD scales, several single-item indicators were administered exclusively to the parental subsample, focusing on relationship satisfaction and dyadic coping. Global satisfaction with the relationship with one’s romantic partner was measured on an 11-point Likert scale (0 = not at all satisfied, 10 = completely satisfied).

The six dyadic-coping items were derived from items 36 and 37 of the Dyadic Coping Inventory (DCI; Bodenmann, 2008), as adapted and validated in Hungarian by Martos et al. (2012). These two DCI items assess satisfaction with partner support and joint coping, and perceived dyadic coping efficacy. For the present study, their wording was minimally adapted to three specific life domains: child-related stress, stress arising within the couple relationship, and everyday stress. This resulted in six context-specific items, with parallel items assessing satisfaction with dyadic coping and its perceived efficacy in each domain. Responses were rated on a 5-point Likert scale (1 = not at all, 5 = completely).

The English wording was aligned with the corresponding English DCI items and adapted to the three life domains as follows: “I am satisfied with the support I receive from my partner and the way we deal with stress related to the children together.”; “I am satisfied with the support I receive from my partner, and I find that, as a couple, the way we deal with stress related to the children together is effective.”; “I am satisfied with the support I receive from my partner and the way we deal with stress arising from our relationship together.”; “I am satisfied with the support I receive from my partner, and I find that, as a couple, the way we deal with stress arising from our relationship together is effective.”; “I am satisfied with the support I receive from my partner and the way we deal with everyday stress together.”; “I am satisfied with the support I receive from my partner, and I find that, as a couple, the way we deal with everyday stress together is effective.”

Each of the six context-specific items was analyzed individually, and no composite score was formed. Accordingly, these adapted items were treated as brief external indicators of parents’ perceived dyadic coping rather than as a separate psychometric scale.

### 2.4. Data Analysis

Analyses were performed in R (version 4.4.1; R Core Team, 2024) via RStudio (version 2025.05.1; Posit Software, PBC, 2025), primarily using lavaan (version 0.7-2), psych (version 2.5.6), lme4 (version 2.0-1), semTools (version 0.5-8), and clubSandwich (version 0.7.0). Before analysis, the six reverse-keyed FAD-GF items were recoded so that higher scores consistently indicated poorer family functioning. There was no item-level missingness across the 12 FAD-GF items or the three embedded BAFFS items; therefore, no case deletion or score prorating was required.

Analyses began with item-level response distributions and descriptive statistics. Within-family dependence was then assessed using item-level intraclass correlation coefficients (ICCs), with family ID as the clustering variable.

CFA indicators were treated as ordered categorical variables and estimated using WLSMV. Total-sample models included family ID as the clustering variable to obtain cluster-adjusted estimates. An explicit multilevel CFA was not fitted because the small and unbalanced cluster sizes and the heterogeneous representation of family roles provided insufficient support for estimating separate within- and between-family measurement structures; cluster-adjusted respondent-level models were therefore used.

Model fit was evaluated jointly using scaled χ^2^, CFI, TLI, RMSEA with 90% CI, and SRMR. CFI and TLI values of 0.90/0.95 and RMSEA values of 0.08/0.06 were used as approximate thresholds for acceptable/good fit; SRMR below 0.08 was considered favorable. Standardized loadings above 0.40 were considered substantively meaningful.

Five candidate FAD-GF structures were examined: the original one-factor model, a general factor with an orthogonal reverse-wording method factor, correlated positive- and negative-wording factors, the FAD-GF6+, and Cong et al.’s (2022) three-factor model. Because none adequately represented the item covariance structure, dimensionality was subsequently explored using minimum-residual EFA with oblimin rotation and polychoric correlations. Parallel analyses used 1000 iterations and the 95th percentile of the simulated and resampled eigenvalues. Analyses were conducted in the total sample and repeated in a sensitivity sample containing one randomly selected respondent per family. Four- and five-factor solutions were compared using loadings, factor correlations, congruence, admissibility, and fit indices. General-factor strength was further examined using unidimensionality indices and Schmid–Leiman transformations, including ωH, ωT, ECV, and item-level p^2^ (the proportion of an item’s common variance attributable to the general factor).

These findings informed exploratory Model 6, which retained one general factor and simultaneously included seven residual covariances supported by recurring CFA modification-index patterns, replicated EFA patterns, and item-content overlap. No further residual covariances were added based on Model 6 modification indices. Accordingly, Model 6 was treated as exploratory rather than independently validated.

FAD-GF measurement invariance was examined using the Model 6 specification, retaining the same seven residual-covariance pairs in each group. For the ordinal invariance analyses, we followed the identification procedure of Wu and Estabrook (2016), as implemented in semtools v0.5-8 and lavaan v0.7-2. Under this procedure, equality of item thresholds is tested first, allowing latent-response intercepts and residual variances in the nonreference group to become separately estimable. This distinction between item thresholds and latent-response intercepts, as well as the cumulative sequence of constraints, is also documented in semTools (Jorgensen et al., 2026). Five levels were therefore examined: configural, threshold, metric (equal loadings), scalar (equal latent-response intercepts), and strict invariance (equal residual variances). Comparisons included parents versus adolescents, fathers versus mothers, index adolescents versus siblings, and ASD versus AN parents. The DM group was excluded from diagnostic invariance testing because of its small parental sample (n = 43), and the index-adolescent–sibling comparison was treated as exploratory. Invariance was primarily supported when decreases in CFI did not exceed 0.010, increases in RMSEA did not exceed 0.015, and increases in SRMR did not exceed 0.030 for metric or 0.010 for threshold, scalar, and strict invariance. Changes in TLI and scaled-and-shifted χ^2^ difference tests were also reported but were not used as sole decision criteria. When full invariance was unsupported, constraint-specific diagnostics were used to examine partial invariance.

The three-item BAFFS was analyzed separately using cluster-adjusted ordinal CFA. Because its one-factor model was just-identified (df = 0), global fit could not test unidimensionality; evaluation therefore focused on standardized loadings and admissibility. Group-specific solutions were inspected before invariance testing. Invariance results were interpreted only when the corresponding configural solutions were admissible. Because configural BAFFS models were necessarily saturated, the first constrained model was evaluated using its absolute fit and scaled difference test rather than change from the perfect configural fit.

Internal consistency was assessed using Cronbach’s α and McDonald’s ωT. Omega total was estimated from a one-factor minimum-residual solution based on Pearson correlations among observed item scores. Its 95% CIs, and those of α, were obtained using 2000 bootstrap samples with resampling at the family level. These ωT estimates were distinct from the polychoric Schmid–Leiman estimates used in the general-factor analysis. AVE was calculated as the mean squared standardized loading and interpreted descriptively relative to 0.50.

FAD-GF and BAFFS scores were calculated as item means. Descriptive statistics were computed across respondent-role and diagnostic groups. External and demographic associations were summarized using Spearman correlations. Their significance was evaluated through regressions of standardized ranks using CR2 family-cluster-robust standard errors and Satterthwaite-adjusted degrees of freedom. No multiplicity correction was applied because these analyses were exploratory. FAD-GF–BAFFS correlations were treated as descriptive part–whole associations because the BAFFS items are included in the FAD-GF.

## 3. Results

### 3.1. Item-Level Descriptive Statistics

Detailed response distributions for all 12 FAD-GF items in the total sample and by family role are reported in Supplementary Table S2. Reverse-scored items were recoded before analysis so that higher values consistently indicated more problematic family functioning. In the total sample, item means ranged from 1.52 to 2.22, and all items showed positive skewness, ranging from slight to marked (0.19–1.51), reflecting a greater concentration of responses in the lower problem categories.

### 3.2. Within-Family Dependence

Before conducting structural analyses, non-independence within family units was examined by calculating Intraclass Correlation Coefficients (ICC) for each FAD item, using the family ID as the clustering variable. ICC values across all 12 items were meaningful, ranging from 0.19 to 0.42, indicating meaningful within-family similarity and supporting the use of cluster-adjusted analytical approaches. Within-family agreement was highest for items FAD51 (ICC = 0.42; “*We don’t get along well together*”) and FAD31 (ICC = 0.39; “*There are lots of bad feelings in the family*”).

### 3.3. Confirmatory Factor Analysis of the General Functioning Scale (FAD-GF)

First, the structural validity of the FAD-GF was examined using cluster-adjusted ordinal confirmatory factor analyses. We evaluated five prespecified models representing solutions previously proposed in or motivated by the FAD-GF literature: the original 12-item one-factor model (Model 1), a one-factor model with an orthogonal reverse-wording method factor (Model 2), a correlated two-factor model separating positively and negatively worded items (Model 3), the six-item FAD-GF6+ model (Model 4), and the previously proposed 11-item three-factor model (Model 5). The original 12-item one-factor model (Model 1) showed poor fit to the data (Table 2). Several large modification indices indicated substantial localized dependencies, most prominently between FAD11 and FAD21 (MI = 82.96), FAD16 and FAD36 (MI = 66.00), and FAD21 and FAD26 (MI = 43.20). The 20 largest modification indices are reported in Supplementary Table S3.

Because the FAD-GF contains equal numbers of positively and negatively worded items, and wording polarity can generate shared method variance and apparent multidimensionality, we first examined whether the misfit was attributable to the originally negatively worded items requiring reverse coding. Model 2 retained the general factor and added an orthogonal method factor for the six reverse-coded items. This model also showed poor fit (Table 2). Although all method-factor loadings were statistically significant, they varied substantially in magnitude and direction (standardized λ = −0.45 to 0.25), providing no evidence of a coherent method effect shared by the reverse-coded items. The largest modification indices continued to indicate both localized residual dependencies and potential method-factor loadings for items outside the designated set (Supplementary Tables S4 and S5).

Model 3 specified two correlated factors separating the positively and negatively worded items. Its fit was similarly poor (Table 2). Moreover, the two factors were very strongly correlated (r = 0.946, SE = 0.013, z = 75.50, *p* < 0.001), providing little support for their interpretation as distinct dimensions. Modification indices suggested numerous residual covariances and cross-loadings rather than a clear separation according to wording polarity (Supplementary Tables S6 and S7).

Model 4 tested the previously proposed six-item positive-wording version of the scale (FAD-GF6+). Although its CFI (0.936) and SRMR (0.047) were more favorable, its TLI (0.893) and RMSEA (0.146, 90% CI [0.114, 0.181]) indicated inadequate overall fit. The standardized loadings were strong (λ = 0.62–0.86), but modification indices continued to identify localized residual dependencies, particularly between FAD16 and FAD36 (MI = 36.15) and between FAD6 and FAD56 (MI = 11.14; Supplementary Tables S8 and S9).

Taken together, the method-factor model, the positive–negative two-factor model, and the positive-item-only model indicate that the poor fit of the original one-factor structure cannot be attributed primarily to reverse coding or wording polarity. Instead, the recurring modification-index patterns suggest localized dependencies among particular subsets of items. Accordingly, we next evaluated an additional previously proposed multidimensional model and subsequently explored the dimensionality of the full item set.

Model 5 tested the 11-item three-factor structure proposed by Cong et al. (2022), comprising negative-functioning, positive-functioning, and emotional-communication factors. Although all standardized factor loadings were substantial (λ = 0.54–0.89), overall model fit remained inadequate: the CFI (0.909) and SRMR (0.054) were comparatively favorable, but the TLI (0.878) and RMSEA (0.118, 90% CI [0.102, 0.133]) were not acceptable (Table 2). The factor correlations were also strong, ranging from 0.70 to 0.90, with the strongest association between the negative- and positive-functioning factors (r = 0.90), indicating limited separation between the proposed dimensions (Supplementary Table S10). Modification indices identified both substantial cross-loadings—most notably FAD6 on the emotional-communication factor (MI = 38.52, standardized EPC = 0.51) and FAD46 on the negative-functioning factor (MI = 17.46, standardized EPC = 0.91)—and several localized residual dependencies (Supplementary Table S11). Thus, the previously proposed three-factor structure did not provide a sufficiently clear or well-fitting representation of the item covariance pattern, prompting an exploratory examination of the scale’s dimensionality.

### 3.4. Dimensionality, General-Factor Strength, and Local Dependence

Because none of the prespecified CFA models adequately represented the item covariance structure, the dimensionality of the full item set was examined exploratorily. Parallel analysis suggested five factors in the total sample (N = 562) and four factors in the sensitivity sample containing one randomly selected respondent per family (n = 213; Supplementary Table S12 and Figure S1). Nevertheless, both analyses were strongly dominated by the first factor: its observed eigenvalue was 6.014 in the total sample and 6.333 in the sensitivity sample, whereas the corresponding second eigenvalues were only 0.619 and 0.522.

The discrepancy concerned the comparatively small secondary factors. The fifth eigenvalue exceeded the resampled reference value in the total sample (0.118 vs. 0.086) but not in the sensitivity sample (0.125 vs. 0.137); in the latter analysis, the third and fourth eigenvalues also exceeded their reference values only marginally. Thus, the precise number of secondary factors was not fully stable across samples.

The four- and five-factor EFA solutions provided mixed evidence of fit. Because the analyses were based on polychoric correlation matrices, their fit indices were interpreted descriptively. CFI (0.951–0.977) and RMSR (0.017–0.027) were favorable, whereas TLI (0.865–0.904) and RMSEA (0.103–0.122) remained inadequate. Within-sample BIC comparisons favored five factors in the total sample but four factors in the sensitivity sample, consistent with the parallel-analysis results (Supplementary Table S13). The factors were moderately to strongly correlated (r = 0.36–0.65), although corresponding factors generally showed high cross-sample congruence (0.92–0.98 for the four-factor solutions and 0.88–0.98 for the five-factor solutions).

Several substantively interpretable patterns recurred across solutions (Table 3; Supplementary Table S14), most clearly an emotional-communication cluster comprising FAD11, FAD21, and FAD26. Additional patterns involved positive support and confiding, acceptance, negative relational climate, and decision making and problem solving. However, these did not support distinct subscales: their composition varied between the four- and five-factor solutions, several items cross-loaded, the acceptance factor was largely dominated by FAD16, and the decision/problem-solving factor was essentially defined by FAD41. The latter two patterns were also associated with boundary or improper communality estimates. Overall, the EFA results suggested recurring local associations around a dominant common dimension rather than four or five clearly separable factors, motivating the subsequent evaluation of general-factor strength and local dependence.

Complementary unidimensionality indices indicated pronounced first-factor dominance in both parallel-analysis-retained solutions. In the total and sensitivity samples, respectively, the unidimensionality index was 0.927 and 0.943, ECV was 0.720 and 0.784, and the first-to-second-factor ratio was 5.691 and 6.731. At the same time, the low one-factor CFIs (0.809 and 0.797) indicated that the item structure was not strictly unidimensional (Table 4; Supplementary Table S15).

Schmid–Leiman transformations were subsequently used to separate variance attributable to the general factor from variance attributable to the residualized group factors. Omega hierarchical was 0.806 in the total sample and 0.798 in the sensitivity sample; relative to omega total, the general factor accounted for 84.2% and 83.7% of the reliable total-score variance, respectively (Table 5). All items had at least moderate general-factor loadings, ranging from 0.449 to 0.821 in the total sample and from 0.484 to 0.806 in the sensitivity sample. The general factor accounted for more than half of the common variance for 9 of the 12 items in the total sample and 11 of the 12 items in the sensitivity sample. FAD41 showed the lowest p^2^ in both analyses (0.304 and 0.405), indicating a comparatively large local component. Expanded general-factor diagnostics are reported in Supplementary Table S16, item-level general-factor loadings and p^2^ coefficients in Supplementary Table S17, and the complete Schmid–Leiman loading matrices in Supplementary Table S18. Because the four-factor sensitivity solution produced an ultra-Heywood estimate involving FAD41, its specific-factor estimates should be interpreted cautiously; nevertheless, the general-factor indices were closely comparable across the two samples.

The residualized group factors made substantially smaller contributions to the reliability of the corresponding factor-defined item subsets: ωgroup ranged from 0.146 to 0.417 in the total sample and from 0.163 to 0.369 in the sensitivity sample (Supplementary Table S19). Moreover, several subsets contained very few items or changed composition across the four- and five-factor solutions. Accordingly, these subsets were interpreted as manifestations of local dependence rather than as sufficiently established subscales. Taken together, the findings supported interpreting the total score as primarily reflecting a general family-functioning dimension, while suggesting that recurring local dependencies contributed to the poor fit of the simple one-factor CFA. We therefore next examined whether explicitly modeling these local dependencies could account for the remaining model misfit.

For this purpose, Model 6 retained a single general factor while allowing seven residual covariances representing the most consistently supported local dependencies. These covariances were selected based on convergence among recurring modification-index patterns across the preceding CFA models, the replicated EFA patterns, and substantive overlap in item content. They comprised all three pairwise residual covariances among FAD11, FAD21, and FAD26; the FAD16–FAD36 and FAD31–FAD51 pairs; and the FAD1–FAD41 and FAD41–FAD46 pairs. The specification was treated as exploratory, and no additional residual covariances were introduced based on the modification indices obtained from Model 6.

The model converged without inadmissible estimates and showed broadly acceptable fit, χ^2^(47) = 105.28, *p* < 0.001, CFI = 0.962, TLI = 0.946, RMSEA = 0.078, 90% CI [0.062, 0.094], and SRMR = 0.034 (Table 2). Compared with the original one-factor model, the CFI increased from 0.815 to 0.962, the TLI from 0.774 to 0.946, the RMSEA decreased from 0.159 to 0.078, and the SRMR from 0.077 to 0.034. Thus, accounting for the specified local dependencies substantially improved the representation of the item covariance structure, although the model should not be interpreted as a separately validated confirmatory structure.

All standardized general-factor loadings were statistically significant and ranged from 0.491 to 0.858 (Table 6). The strongest loadings were observed for FAD56 (λ = 0.858) and FAD51 (λ = 0.810), whereas FAD1 (λ = 0.491; “*Planning family activities is difficult because we misunderstand each other.*”) had the lowest loading. The semantic difference between the original item and the Hungarian wording administered in the present study is considered further in the Discussion. The seven standardized residual correlations were positive and statistically significant, ranging from 0.275 to 0.563. The largest residual associations occurred within the emotional-communication cluster comprising FAD11, FAD21, and FAD26 (r_res_ = 0.438–0.563). The residual correlation was also substantial between the two acceptance items, FAD16 and FAD36. More moderate localized associations were observed between FAD41 and FAD46, FAD1 and FAD41, and FAD31 and FAD51. These residual correlations closely reflected the recurring patterns identified in the preceding CFA and EFA results.

Taken together, Model 6 reconciled the poor fit of the simple one-factor CFA with the evidence of general-factor dominance. Although strict local independence was not supported, all items remained substantially associated with the general factor after the recurring local dependencies were modeled. The findings therefore support interpreting the FAD-GF total score as an indicator of overall family functioning in this sample, while the localized item associations should not be interpreted as sufficiently established separate subscales.

### 3.5. Measurement Invariance Analysis of the General Functioning Scale (FAD-GF) Across Generational Roles

To determine whether the FAD-GF scale operates equivalently for parents and adolescents, a sequence of increasingly restrictive multigroup confirmatory factor analyses was estimated using cluster-adjusted WLSMV (Table 7). All FAD-GF measurement-invariance analyses were based on the exploratory Model 6 specification and retained the same seven residual covariances in each group. The invariance findings should therefore be interpreted as conditional on this exploratory measurement model.

The configural model showed good fit (CFI = 0.987, RMSEA = 0.054), establishing a baseline. Threshold and metric invariance were supported by negligible changes in the approximate fit indices (|ΔCFI| ≤ 0.001 and |ΔRMSEA| ≤ 0.004), although the scaled chi-square difference test for metric invariance was significant (*p* = 0.040). The scalar invariance model, however, resulted in a deterioration in fit (ΔCFI = −0.011, ΔRMSEA = 0.014), indicating that full scalar invariance was not supported.

To achieve partial scalar invariance, equality constraints on the latent-response intercepts of three items were released sequentially. For FAD16 (“*Individuals are accepted for what they are*”) and FAD36 (“*We feel accepted for what we are*”), the estimated latent-response intercepts were higher among parents, indicating a greater propensity to select higher, more problematic response categories at the same level of the general family-functioning factor. This indicates that parents perceived less mutual acceptance within the family than adolescents did. In case of FAD56 (“*We confide in each other*”), the latent-response intercept was higher among adolescents, suggesting that adolescents perceived a lower level of mutual confidence or confiding within the family unit than the parental generation. Thus, the localized noninvariance concerned acceptance-related items among parents and confiding among adolescents. The partial scalar model (releasing intercepts for items FAD16, FAD36, and FAD56) showed good fit (CFI = 0.986, RMSEA = 0.049), with negligible changes relative to the metric model (ΔCFI = −0.001, ΔRMSEA = 0.000).

Finally, strict invariance of the residual variances was tested within the partial scalar model by constraining all residual variances to equality while retaining the three freely estimated intercepts. The resulting model also showed good fit (CFI = 0.986, RMSEA = 0.046), with negligible changes relative to the partial scalar model (ΔCFI = 0.000, ΔRMSEA = −0.003); the scaled chi-square difference test was nonsignificant (*p* = 0.059). These findings support configural, threshold, metric, and partial scalar invariance, together with invariant residual variances, across parents and adolescents.

### 3.6. Measurement Invariance Analysis of the General Functioning Scale (FAD-GF) Between Paternal and Maternal Roles

Measurement invariance was examined to ensure that the FAD-GF scale operates consistently across parental roles. The hierarchical testing procedure (see Table 7) demonstrated stable fit across groups; the configural model showed good fit (CFI = 0.989, RMSEA = 0.057), and changes in fit indices remained within recommended thresholds at the threshold (ΔCFI = −0.001, ΔRMSEA = −0.002), metric (ΔCFI = 0.001, ΔRMSEA = −0.006), scalar (ΔCFI = 0.002, ΔRMSEA = −0.006), and strict levels (ΔCFI = −0.005, ΔRMSEA = 0.008). Although the scaled chi-square difference test for strict invariance was significant (*p* = 0.001), the changes in the approximate fit indices did not exceed the prespecified thresholds. These results support threshold, metric, scalar, and strict invariance across fathers and mothers according to the prespecified approximate-fit criteria, although the significant strict-level chi-square difference test warrants some caution.

### 3.7. Measurement Invariance Analysis of the General Functioning Scale (FAD-GF) Between Index Adolescents and Siblings

To examine whether the FAD-GF operated equivalently among index adolescents and siblings, an exploratory multigroup invariance analysis compared 126 index adolescents with 70 siblings (Table 7). The configural model yielded favorable CFI and TLI values (CFI = 0.972, TLI = 0.960) and an acceptable SRMR (0.067), although the RMSEA (0.084, 90% CI [0.060, 0.106]) slightly exceeded the prespecified criterion. Baseline fit was therefore mixed rather than unequivocally acceptable. Imposing equality constraints did not produce deterioration in approximate model fit. Threshold invariance (ΔCFI = 0.002, ΔRMSEA = −0.007), metric invariance (ΔCFI = 0.003, ΔRMSEA = −0.007), scalar invariance (ΔCFI = 0.002, ΔRMSEA = −0.006), and strict invariance (ΔCFI = 0.001, ΔRMSEA = −0.004) were all supported according to the prespecified change criteria. The scaled chi-square difference tests were also nonsignificant at every step (*p* = 0.178–0.894). The final strict model showed improved approximate fit relative to the configural model (CFI = 0.979, RMSEA = 0.059). Thus, this sensitivity analysis provided no indication of substantial measurement noninvariance between index adolescents and siblings. However, because the sibling group was relatively small, the findings should be regarded as preliminary and interpreted cautiously.

### 3.8. Measurement Invariance Analysis of the General Functioning Scale (FAD-GF) Across Clinical Diagnostic Groups

Finally, measurement invariance of the FAD-GF scale was tested across the parental samples of the ASD and AN groups (Table 7). Given the limited size of the DM parental sample (n = 43), diagnostic-group invariance testing was restricted to the ASD (n = 146) and AN (n = 177) groups. The configural model showed good fit (CFI = 0.985, RMSEA = 0.056), providing a suitable baseline for invariance testing. Threshold invariance was supported by negligible changes in fit (ΔCFI = 0.000, ΔRMSEA = −0.003). Metric invariance was similarly supported (ΔCFI = −0.004, ΔRMSEA = 0.003), although the scaled chi-square difference test was significant (*p* = 0.015). Full scalar invariance was also supported because the changes in approximate fit remained within the prespecified criteria (ΔCFI = −0.005, ΔRMSEA = 0.005). Although the corresponding scaled chi-square difference test was significant (*p* = 0.002), no item intercepts needed to be released. Consequently, latent mean comparisons between the ASD and AN groups are supported under the full scalar model. Strict invariance was not supported: the decrease in CFI (ΔCFI = −0.013) and increase in SRMR (ΔSRMR = 0.017) exceeded the prespecified criteria, and the scaled chi-square difference test was significant (*p* < 0.001). Thus, equality of the item residual variances could not be assumed across the ASD and AN groups. This finding does not invalidate scalar invariance or latent mean comparisons, but it indicates that item-specific residual variances may differ between the two diagnostic groups.

### 3.9. Confirmatory Factor Analysis of the Brief Assessment of Family Functioning Scale (BAFFS)

The internal structure of the three-item BAFFS, comprising FAD26, FAD51, and FAD56, was examined using cluster-adjusted ordinal CFA. Because a one-factor model with three indicators is just-identified (df = 0), its necessarily perfect global fit cannot be used to test unidimensionality. The total-sample solution was nevertheless admissible, and all three standardized loadings were statistically significant: 0.640 for FAD26, 0.803 for FAD51, and 0.870 for FAD56 (all *p* < 0.001; Table 6). These estimates indicate that each item was substantially associated with the specified common factor, although they provide descriptive evidence rather than an independent test of the hypothesized dimensional structure.

Group-specific diagnostics and ordinal measurement-invariance analyses were conducted to examine whether this three-item representation behaved similarly across respondent groups (Supplementary Tables S20 and S21). The group-specific solutions were admissible for fathers, mothers, and parents in both the ASD and AN groups. Across fathers and mothers, threshold, metric, and scalar invariance were supported, whereas strict invariance was not. Across ASD and AN parents, threshold and metric invariance were supported, but full scalar invariance was not: although ΔCFI remained within the prespecified criterion (−0.008), the changes in TLI (−0.013), RMSEA (0.057), and SRMR (0.021), together with the significant scaled chi-square difference test (*p* = 0.020), indicated deterioration in fit.

In contrast, the separate adolescent and sibling solutions produced an inadmissible negative residual variance for FAD56, and its residual variance was close to the boundary in the index-adolescent group. The pairwise polychoric correlations nevertheless remained moderate to strong across these groups, indicating that the problem did not reflect an absence of association among the three items. Rather, their relative covariance pattern differed, with FAD56—and particularly its association with FAD51—receiving greater weight in the adolescent groups. Measurement-invariance results involving adolescents and siblings were therefore not interpreted. Overall, the findings support retaining the BAFFS as a brief observed index for descriptive and association analyses, particularly in parental reports, but they do not establish it as a uniformly unidimensional and measurement-invariant latent scale across respondent roles. Comparisons involving adolescent and sibling BAFFS scores should consequently be interpreted cautiously and require further validation in larger, more balanced samples.

### 3.10. Internal Consistency and Average Variance Extracted

Internal consistency coefficients for the 12-item FAD-GF and 3-item BAFFS are summarized in Table 8. Average variance extracted (AVE) was calculated from the standardized loadings of the respective total-sample CFA models. The FAD-GF scale demonstrated high internal consistency in the total sample (α = 0.893, ωT = 0.896) and all subgroups (α = 0.876–0.907; ωT = 0.886–0.909). The AVE was 0.484, slightly below the conventional 0.50 threshold, indicating that the general factor accounted, on average, for slightly less than half of the variance in the individual indicators. Although the high ωT supports the consistency of the total score, it does not eliminate this limitation of the item-level variance decomposition.

The BAFFS showed acceptable internal consistency relative to its brevity (α = 0.747, ωT = 0.756 in the total sample), with subgroup estimates ranging from 0.687 to 0.785 for α and from 0.693 to 0.796 for ωT. Its AVE was 0.604. However, because the three-indicator CFA was just-identified, this value is a descriptive function of the estimated loadings and cannot independently confirm unidimensionality or structural validity. The broadly similar internal-consistency estimates provide descriptive evidence regarding score consistency in the present sample but do not, by themselves, establish measurement invariance or generalizability across diverse Hungarian populations.

### 3.11. Descriptive Statistics and External Associations

Descriptive statistics and the correlation matrix for the FAD-GF and BAFFS scores are presented in Table 9. A positive skew reflected the clinical sample nature, where most families reported healthy functioning and a smaller subset scored in the dysfunctional range.

The BAFFS scores were strongly correlated with the 12-item FAD-GF scale (*r_s_* = 0.85 to 0.87) across all subgroups. However, because the three BAFFS items are included in the 12-item FAD-GF, these are part–whole correlations and are reported descriptively rather than interpreted as independent evidence of convergent validity.

Both scales exhibited expected patterns with external constructs: higher family dysfunction was generally associated with higher perceived stress (PSS-4: *r_s_* = 0.26 to 0.44) and lower subjective well-being (WHO-WBI: *r_s_* = −0.11 to −0.44). Notably, well-being associations were strongest in the DM group and non-significant in the AN group. The magnitude of these associations varied descriptively across the diagnostic groups, but differences between the group-specific correlation coefficients were not formally tested.

Age did not correlate with perceived family functioning. Additionally, regarding family size, a weak but significant negative correlations with family dysfunction was observed within the adult group (*r_s_* = −0.12 to −0.13), suggesting that larger family size was associated with slightly better perceived functioning. Given the small magnitude of these coefficients, they should be interpreted cautiously.

Both scales showed negative associations with relationship satisfaction and dyadic coping. These associations were strongest in the ASD group, where relationship satisfaction (*r_s_* = −0.72) and perceived efficacy in managing everyday stress (*r_s_* = −0.62) were most closely tied to global family health. Conversely, these associations were descriptively weakest in the AN group. In most instances, perceived dyadic coping efficacy consistently shared stronger associations with family functioning than emotional satisfaction with the coping process.

Because a large number of exploratory correlations were examined, the resulting *p*-values should therefore be interpreted cautiously, with emphasis placed on the magnitude and consistency of the associations rather than on isolated significance tests.

## 4. Discussion

### 4.1. Psychometric Performance of the FAD-GF and BAFFS

The present study examined the psychometric properties of the Hungarian FAD-GF and BAFFS in a multi-informant clinical sample of families of adolescents aged 12–18 years. The findings support cautious interpretation of the FAD-GF total score in this sample, although its internal structure proved more complex than a strictly unidimensional model would imply. Evidence for the BAFFS was more limited, particularly with respect to its latent structure and its use across adolescent respondent groups.

None of the prespecified FAD-GF models based on solutions previously proposed in the literature provided an adequate representation of the item covariance structure. The original one-factor model fitted poorly, and this misfit was not resolved by introducing a reverse-wording method factor, separating positively and negatively worded items, testing the six-item FAD-GF6+ (Boterhoven de Haan et al., 2015), or applying the previously proposed 11-item three-factor structure (Cong et al., 2022). This variability is consistent with the broader FAD-GF literature, in which different factor structures and item selections have received support across populations and linguistic contexts (Boterhoven de Haan et al., 2015; Babar et al., 2021; Badovinac et al., 2024; Assaf et al., 2025). In the present sample, the alternative models did not yield sufficiently distinct and well-fitting substantive dimensions to justify interpreting them as separate components of family functioning.

The exploratory analyses provided a more differentiated account of the scale structure. Although parallel analysis and EFA identified several secondary item patterns, the first factor was clearly dominant, while the smaller factors were less stable across the full sample and the one-respondent-per-family sensitivity analysis. The general-factor analyses likewise showed that a substantial proportion of reliable total-score variance was attributable to a common family-functioning dimension. Alongside this general factor, several local item dependencies recurred across analyses, particularly among items concerning emotional communication, acceptance, negative relational climate, and decision making. Thus, the findings are compatible with a dominant general family-functioning factor accompanied by recurring local dependencies, rather than with a simple one-factor structure satisfying strict local independence.

Exploratory Model 6 was developed to represent this pattern by estimating seven residual correlations simultaneously in addition to the general factor. Its fit was broadly acceptable, but the model was derived from the present sample. The residual dependencies were selected from converging CFA, EFA, general-factor, and item-content evidence, and no further residual correlations were introduced after Model 6 had been specified. Accordingly, the model should be regarded as an exploratory measurement specification that requires independent replication. Its main contribution in the present analysis is to show that the pronounced general-factor signal can coexist with systematic local associations among particular items.

The reliability findings also support a qualified interpretation of the FAD-GF total score. Internal consistency was high in the full sample (α = 0.893, ωT = 0.896), whereas the AVE was 0.484. The latter remains slightly below the conventional 0.50 criterion (Fornell & Larcker, 1981), indicating that the general factor accounted, on average, for slightly less than half of the variance in the individual indicators. The high internal-consistency estimates therefore should not be taken to eliminate the qualification introduced by the AVE and the observed local dependencies. In this sample, the total score can be interpreted as a broad indicator of perceived family functioning, while the structural evidence does not support an unequivocally unidimensional representation of the item set.

FAD1 had the lowest general-factor loading in the final exploratory model and also warrants consideration from a translation perspective. The original English item refers to difficulties in planning family activities because family members “misunderstand each other” (Epstein et al., 1983), whereas the Hungarian wording administered in the present study places greater emphasis on disagreement. Although these meanings overlap, disagreement does not necessarily imply misunderstanding and may therefore shift the emphasis of the item away from the communication process expressed in the original wording. In light of its comparatively low loading and this semantic difference, the Hungarian translation of FAD1 was reviewed in consultation with colleagues involved in the original adaptation. A slightly revised Hungarian wording was agreed upon and is included in the publicly available version recommended for future use. The present data, however, were collected using the earlier Hungarian wording. The evidence obtained for the three-item BAFFS was more limited. Previous studies have supported its use as an ultra-brief measure of global family functioning (Mansfield et al., 2019; Spitzer et al., 2022), but several features of the present analysis constrain the conclusions that can be drawn from this sample. The one-factor CFA was just-identified and therefore could not provide an empirical test of unidimensionality or global model fit. Its AVE should consequently be treated as a descriptive function of the standardized loadings rather than as evidence establishing structural validity. Similarly, the strong association between the BAFFS and FAD-GF cannot provide independent evidence of convergent validity because all three BAFFS items are contained within the 12-item FAD-GF. Group-specific analyses also produced inadmissible or near-boundary solutions involving adolescent respondents, which further limits conclusions about the scale across family roles.

The brevity of the BAFFS may nevertheless make it useful as an observed index when minimizing respondent burden is important, particularly in parental reports, consistent with the rationale underlying its original development (Mansfield et al., 2019). The present findings do not establish its suitability for screening, repeated monitoring, or outcome assessment, as these applications would require additional structural and longitudinal evidence. Within the current sample, the FAD-GF has a stronger psychometric basis as a broad measure of perceived family functioning, whereas interpretation of the BAFFS should remain more circumscribed pending further validation.

### 4.2. Measurement Invariance and Multi-Informant Interpretation

The measurement-invariance analyses provided a more differentiated picture of score comparability across family roles and clinical groups. For the FAD-GF, partial scalar invariance was supported across parents and adolescents, strict invariance across fathers and mothers, and preliminary strict invariance across index adolescents and siblings. Across parental diagnostic groups, scalar but not strict invariance was supported between the ASD and AN samples. These findings should be interpreted as conditional on the exploratory Model 6 specification, because the same seven residual covariances were retained throughout the FAD-GF multigroup analyses.

The parent–adolescent comparison indicated that most measurement parameters were sufficiently comparable across generations, although full scalar invariance was not achieved. The localized noninvariance involved FAD16 and FAD36, both concerning acceptance within the family, and FAD56, which concerns mutual confiding. At the same level of the general family-functioning factor, parents showed a greater tendency to endorse more problematic responses on the two acceptance items, whereas adolescents showed a greater tendency to endorse more problematic responses on the item concerning confiding. Informant discrepancies of this kind are not unusual in assessments of child and family functioning and may contain clinically relevant information rather than representing measurement error alone (De Los Reyes & Kazdin, 2005; De Los Reyes, 2011; De Los Reyes et al., 2013). Previous FAD research has likewise documented systematic differences between parental and adolescent perspectives on family functioning (Sawyer et al., 1988; Bagley et al., 2001). The particular pattern observed here is also compatible with developmental changes in privacy, autonomy, self-disclosure, and parent–adolescent communication during adolescence (Branje et al., 2013), although the present cross-sectional data do not establish these developmental processes as the source of the item-level differences.

The father–mother comparison yielded the strongest evidence of measurement equivalence for the FAD-GF, with invariance supported through the strict level according to the prespecified approximate-fit criteria. This increases confidence that observed differences between maternal and paternal FAD-GF scores are less likely to arise from systematic differences in how the items function across parental roles. It does not imply that mothers and fathers necessarily perceive family functioning in the same way. Previous research using the FAD has shown both shared and respondent-specific components in family members’ reports (Georgiades et al., 2008), and maternal and paternal ratings may differ even when the instrument itself operates comparably across parents (Cooke et al., 2015). Collecting separate reports from both parents may therefore provide information that would be lost by treating parental perspectives as interchangeable.

The exploratory comparison between index adolescents and siblings provided no clear indication of substantial FAD-GF measurement noninvariance, with equality constraints supported through the strict level. This result should nevertheless be considered preliminary. The sibling group was comparatively small, and the configural model showed mixed baseline fit, with favorable CFI, TLI, and SRMR but an RMSEA slightly above the prespecified criterion. The findings therefore provide initial support for comparability of FAD-GF scores between index adolescents and their participating siblings, but replication in larger sibling samples is needed before stronger conclusions can be drawn.

Diagnostic-group invariance of the FAD-GF was examined only among parents in the ASD and AN groups because the DM parental sample was too small for sufficiently stable multigroup CFA. Scalar invariance was supported between ASD and AN parents, indicating that latent mean comparisons are tenable under the measurement specification used here, whereas strict invariance was not supported because residual variances differed across groups. Invariance at the scalar level is sufficient for comparisons of latent means, while strict invariance imposes the additional requirement of equal residual variances (Vandenberg & Lance, 2000; Wu & Estabrook, 2016). No between-group differences in latent variances were tested in the revised analyses, and the present results therefore do not provide evidence that family-functioning scores are more heterogeneous in one diagnostic group than in another.

The BAFFS showed a narrower pattern of cross-group comparability. Among fathers and mothers, threshold, metric, and scalar invariance were supported, but strict invariance was not. Among ASD and AN parents, invariance was supported only through the metric level; scalar invariance was not established. Accordingly, the BAFFS items showed comparable relations to the underlying factor across these two diagnostic groups, but latent mean comparisons cannot be assumed to be measurement-equivalent. Comparisons involving adolescents and siblings were not interpreted because the corresponding group-specific solutions included inadmissible or near-boundary estimates for FAD56. Together with the structural limitations described above, this pattern supports a cautious use of the BAFFS as a brief observed index, particularly in parental reports, while providing less support for its use in latent cross-group comparisons.

### 4.3. External Validity Associations and Clinical Patterns

The external associations were generally consistent with the expected direction of relationships between perceived family functioning and broader aspects of psychological and relational adjustment. Higher FAD-GF and BAFFS scores, indicating poorer perceived family functioning, were associated with higher perceived stress and with lower psychological well-being, relationship satisfaction, and perceived dyadic coping efficacy. These findings are compatible with previous evidence linking family functioning and family relationships with psychological well-being and adjustment (Staccini et al., 2014; Thomas et al., 2017). Given the overlap between family functioning and these broader domains, however, the present associations are more appropriately regarded as expected external-construct relations than as independent evidence of convergent validity.

The magnitude of several associations varied descriptively across the ASD, AN, and DM groups. These group-specific patterns should not be interpreted as evidence of diagnostic differences in the relationships between family functioning and the external variables, because differences between the correlation coefficients were not formally tested. The subgroup analyses were exploratory, and no correction for multiple comparisons was applied. Interpretation should therefore focus on the overall direction and consistency of the associations rather than on isolated differences in statistical significance or the relative magnitude of individual subgroup coefficients. Diagnosis-specific patterns may provide hypotheses for future studies designed to compare these associations directly, but the present data do not support explanatory conclusions about why particular relationships appeared stronger or weaker in one clinical group than in another.

This caution is particularly relevant to the DM group. The diabetes sample comprised only 37 families and 43 parental reports, resulting in substantially less precise subgroup estimates than in the ASD and AN parental samples. Associations observed in this group should therefore be considered descriptive and hypothesis-generating. For the same reason, the gender association observed within the DM subgroup is not interpreted further. More generally, the current analyses do not establish condition-specific profiles of family functioning; their contribution is to show that the FAD-GF, and more tentatively the BAFFS, relate to theoretically relevant external constructs across a heterogeneous clinical sample.

From a clinical perspective, the findings support consideration of the FAD-GF as a broad indicator of perceived family functioning in pediatric settings where a global assessment is appropriate. A multi-informant approach may be particularly useful when different family members’ perspectives are clinically relevant, as the preceding invariance analyses indicate that substantial score comparability can coexist with localized informant differences. The present study did not directly compare the FAD-GF with alternative family-functioning instruments, however, and therefore cannot establish that it is superior to other available measures. Its value in the current context lies in the combination of brevity, a broadly interpretable total score, and applicability across several family roles, within the psychometric qualifications described above.

### 4.4. Strength, Limitations, and Future Directions

The present study has several strengths. It examined 562 respondents from 213 families and included mothers, fathers, index adolescents, and adolescent siblings, allowing family functioning to be studied from multiple perspectives within the same clinical context. The sample was drawn from healthcare settings and included families affected by neurodevelopmental, eating, and chronic somatic conditions. Methodologically, the analyses used ordinal cluster-adjusted estimation, examined several prespecified structural models, supplemented CFA with exploratory and general-factor analyses, and evaluated measurement invariance across respondent roles and selected clinical groups. This combination provided a more detailed assessment of score interpretation than would have been possible from internal consistency or single-informant data alone.

The main psychometric qualification concerns the exploratory nature of the final FAD-GF measurement specification. The final model was derived from patterns observed in the present sample, and its seven residual dependencies require replication in independent samples. The FAD-GF invariance findings are also conditional on this exploratory specification. For the BAFFS, the just-identified three-indicator model provided only limited evidence regarding latent structure, and inadmissible or near-boundary solutions restricted interpretation in adolescent respondent groups. The unequal diagnostic-group sizes add further uncertainty to subgroup estimates, particularly in the DM group, which comprised 37 families and only 43 parental reports. Diagnostic-group findings involving this subsample should consequently be regarded as exploratory.

The external-association analyses also have several limitations. Relationship satisfaction and the stress-management indicators (measuring specifically dyadic coping) were available only for parental respondents, limiting conclusions regarding corresponding associations in adolescents. In this study, the six dyadic coping items and the relationship-satisfaction indicator were used as single-item measures, and all external constructs were assessed by self-report. Shared method variance may therefore have contributed to some of the observed associations. The cross-sectional design further prevents conclusions about temporal or causal relationships between perceived family functioning and stress, well-being, or relational adjustment. Because the external-association analyses were exploratory, no multiplicity correction was applied, and differences between diagnostic-group correlation coefficients were not formally tested. These findings are therefore most appropriately interpreted in terms of the overall magnitude, direction, and consistency of the observed external-construct associations.

The age range of the present sample also limits generalizability. The index participants were adolescents aged 12–18 years, and the current findings therefore do not establish the applicability of either measure in families of younger children. Previous research suggests that the FAD may be more challenging to use reliably with children under 12 years of age and that simplified, more structured item wording may be needed in this age group (Bihun et al., 2002). Validation across earlier developmental periods would therefore be valuable, particularly because the meaning and reporting of family relationships may differ with age and respondent role. The present baseline data likewise cannot establish either the FAD-GF or the BAFFS as an outcome measure. Such use would require further evidence on test–retest reliability, responsiveness to clinical change, and the interpretation of clinically meaningful change. Predictive and discriminant validity also remain to be established. These properties will be important for evaluating the potential use of either measure in longitudinal monitoring or treatment-outcome research. Future studies should also examine the performance of the FAD-GF in families facing other pediatric health conditions or other sources of family stress.

Although cluster-robust estimation accounted for within-family dependence in statistical inference, it did not model separate within-family and between-family measurement structures. Given the small and unbalanced cluster sizes and the heterogeneous representation of family roles (Figure 1; Supplementary Materials A, Table S1), the data provided limited support for a role-specific multilevel measurement model. Future studies with larger and more balanced multi-informant family samples could examine such models explicitly.

Additionally, because center-level screening and recruitment records could not be linked to the study dataset, the numbers of eligible and approached families and family members, as well as the numbers declining participation, could not be determined; consequently, recruitment response rates and potential participation bias could not be evaluated.

Questionnaires were completed either online or on paper; however, because the original administration mode was not retained at the respondent level in the dataset supplied to the research team, mode effects could not be examined.

## 5. Conclusions

The present study provides support for the use of the Hungarian FAD-GF total score as a broad indicator of perceived family functioning in this multi-informant clinical sample of families of adolescents. Its structure was characterized by a dominant general factor together with recurring local item dependencies, and the exploratory measurement specification identified here requires independent replication. The FAD-GF also showed substantial cross-group comparability across family roles and between ASD and AN parents, although some forms of invariance were only partial.

Evidence for the BAFFS was more limited. In the present sample, it is best regarded as a brief observed index, particularly for parental reports, rather than as a fully established latent measure across respondent groups. Further structural and longitudinal validation is needed before stronger conclusions can be drawn regarding its use for screening, repeated monitoring, or outcome assessment.

## Figures and Tables

**Figure 1:**
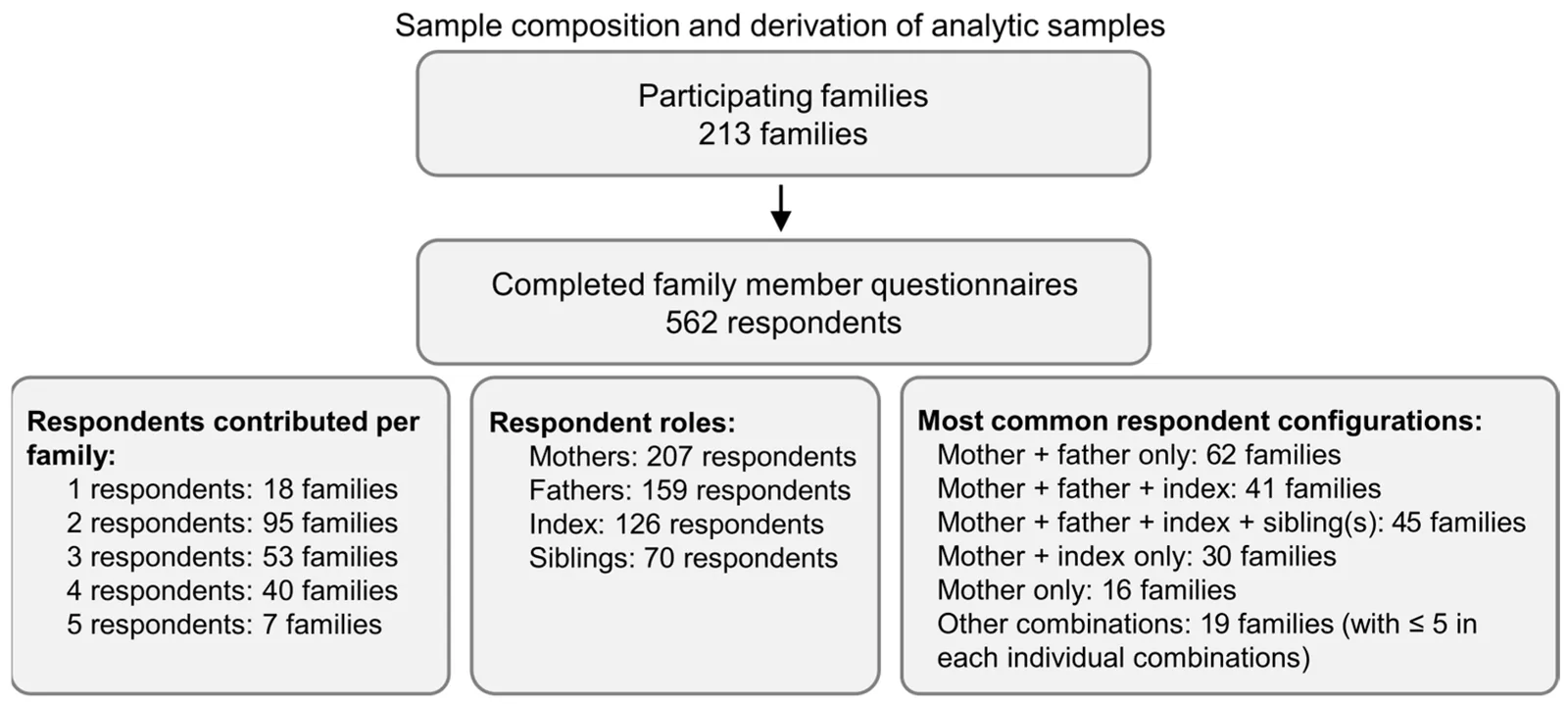
Participant flow and respondent composition within families. Note. The index adolescent was the family member whose condition defined the diagnostic group; sibling(s) refers to participating adolescent siblings. Respondent configurations indicate the family members from whom questionnaires were received and do not necessarily represent household composition. Each configuration included under “Other configurations” occurred in four or fewer families.

**Table 1:** Demographic characteristics of the sample categorized by diagnostic group and family role.

Demographic Characteristics		Autism Spectrum Disorder			Anorexia Nervosa			Diabetes Mellitus		
		Fathers	Mothers	Adolescents	Fathers	Mothers	Adolescents	Fathers	Mothers	Adolescents
*N*		69	77	22	82	95	142	8	35	32
Gender *N (%)*										
	Men			12 (54.5%)			36 (25.4%)			13 (40.6%)
	Women			10 (45.5%)			106 (74.6%)			19 (59.4%)
Age *M(SD)*		42.7 (6.6)	41.5 (6.7)	14.5 (1.7)	48.9 (4.2)	47.4 (4)	15.2 (1.7)	46.6 (6.6)	44.9 (6.8)	15.1 (1.9)
Age groups *N (%)*										
	12			2 (9.1%)			12 (8.5%)			6 (18.8%)
	13			3 (13.6%)			14 (9.9%)			1 (3.1%)
	14			8 (36.4%)			20 (14.1%)			5 (15.6%)
	15			4 (18.2%)			27 (19.0%)			5 (15.6%)
	16			2 (9.1%)			35 (24.6%)			5 (15.6%)
	17			1 (4.5%)			19 (13.4%)			8 (25.0%)
	18			2 (9.1%)			15 (10.6%)			2 (6.3%)
	29–40	27 (39.1%)	40 (51.9%)		2 (2.4%)	4 (4.2%)		2 (25%)	7 (20.0%)	
	41–50	31 (44.9%)	26 (33.8%)		55 (67.1%)	73 (76.8%)		4 (50%)	22 (62.9%)	
	51–64	11 (15.9%)	11 (14.3%)		25 (30.5%)	18 (18.9%)		2 (25%)	6 (17.1%)	
Education *N (%)*										
	Primary	3 (4.3%)	2 (2.6%)		1 (1.2%)	0 (0.0%)		0 (0%)	1 (2.9%)	
	Secondary	24 (34.8%)	26 (33.8%)		34 (41.5%)	33 (34.7%)		5 (62.5%)	19 (54.3%)	
	Higher	41 (59.4%)	49 (63.6%)		47 (57.3%)	62 (65.3%)		3 (37.5%)	15 (42.9%)	
Residence *N (%)*										
	Capital	41 (59.4%)	47 (61.0%)		38 (46.3%)	44 (46.3%)		0 (0%)	0 (0.0%)	
	City/Town	24 (34.8%)	26 (33.8%)		34 (41.5%)	41 (43.2%)		7 (87.5%)	28 (80.0%)	
	Village	4 (5.8%)	4 (5.2%)		10 (12.2%)	10 (10.5%)		1 (12.5%)	7 (20.0%)	

***Note*.** *N* = 562 individual participants from 213 families. Higher education includes university-level degrees and postgraduate (PhD/DLA) qualifications. Percentages are calculated based on the sample size of each specific role within the diagnostic group.

**Table 2:** Model-fit indices for prespecified alternative FAD-GF measurement models based on prior literature.

Models	χ^2^	df	*p*	CFI	TLI	RMSEA	90% CI of RMSEA	SRMR
Model 1	536.97	54	<0.001	0.815	0.774	0.159	[0.146, 0.173]	0.077
Model 2	443.90	48	<0.001	0.856	0.802	0.149	[0.135, 0.164]	0.067
Model 3	522.15	53	<0.001	0.820	0.775	0.159	[0.145, 0.173]	0.076
Model 4	75.88	9	<0.001	0.936	0.893	0.146	[0.114, 0.181]	0.047
Model 5	235.86	41	<0.001	0.909	0.878	0.118	[0.102, 0.133]	0.054
Model 6	105.28	47	<0.001	0.962	0.946	0.078	[0.062, 0.094]	0.034

***Note*.** *N* = 562 (213 clusters). The χ^2^, CFI, TLI, and RMSEA values are scaled or robust estimates from cluster-adjusted WLSMV analyses. Model 1 = original one-factor model; Model 2 = general factor with an orthogonal reverse-wording method factor; Model 3 = correlated positive- and negative-wording factors; Model 4 = one-factor FAD-GF6+; Model 5 = Cong et al.’s (2022) three-factor model. Model 6 was an exploratory one-factor model allowing seven residual covariances identified from converging modification-index patterns, exploratory factor-analytic results, and item-content overlap.

**Table 3:** Recurring Local Item Patterns Across the Four- and Five-Factor Exploratory Solutions.

Recurring Pattern	Items Principally Involved	Evidence Across Solutions	Limitation as a Separate Subscale
Emotional communication	FAD11 ^a^, FAD21 ^a^, FAD26	Formed a distinct three-item cluster in all four solutions, with primary loadings ranging from 0.53 to 0.97 and cross-sample congruence of 0.97–0.98.	The most clearly defined specific cluster, but it remained correlated with all other factors and contained only three items.
Positive support and confiding	FAD6, FAD46, FAD56	Merged with the acceptance items in the four-factor solutions but formed a narrower cluster in the five-factor solutions.	Its composition depended on the number of extracted factors, and several items showed cross-loadings.
Acceptance	FAD16, FAD36	Included in the broader positive factor in the four-factor solutions but separated in the five-factor solutions.	The separated factor was dominated by FAD16 (λ = 0.98–1.02), whereas FAD36 loaded more moderately (λ = 0.33–0.44), producing a near-boundary solution.
Negative relational climate	FAD31 ^a^, FAD51 ^a^, with a secondary contribution from FAD1 ^a^	Recurred in all four solutions, with cross-sample congruence of 0.92–0.98.	Primarily defined by two items, with FAD1 also loading on the decision/problem-solving pattern.
Decision and problem solving	FAD41 ^a^, with secondary contributions from FAD1 ^a^ and FAD46	Recurred across both factor numbers and samples, with FAD41 loading between 0.92 and 0.99.	Essentially dominated by a single item and associated with boundary or improper communality estimates.

***Note*.** The pattern labels are descriptive and were assigned post hoc to facilitate comparison; they do not denote validated subscales. FAD6 consistently showed a secondary loading on the emotional-communication factor (λ = 0.21–0.39) but loaded more strongly on the positive-functioning factor (λ = 0.46–0.70). Superscript ^a^ denotes items that were reverse-scored before analysis.

**Table 4:** Unidimensionality and First-Factor Dominance in the Parallel-Analysis-Retained Solutions.

Sample	Retained Factors	u	τ	ρc	ECV	F1/F2	MAP	One-Factor CFI
Total sample	5	0.927	0.949	0.976	0.720	5.691	0.037	0.809
Sensitivity sample	4	0.943	0.962	0.981	0.784	6.731	0.039	0.797

***Note*.** The number of factors corresponds to the result of the parallel analysis in each sample. u = unidimensionality index, calculated as the product of τ and ρc; τ = tau-equivalence component; ρc = congeneric-fit component and should not be interpreted as composite reliability; ECV = explained common variance of the first unrotated factor; F1/F2 = ratio of the first to second factor; MAP = minimum average partial statistic. The four-factor sensitivity solution produced an ultra-Heywood warning involving FAD41; statistics derived from its specific-factor structure should therefore be interpreted cautiously. Total sample: N = 562. The sensitivity sample contained one randomly selected respondent from each family (*n* = 213).

**Table 5:** General-Factor Strength Across the Four- and Five-Factor Schmid–Leiman Solutions.

Sample	Retained Factors	ωH	ωT	ωH/ωT	ECVG	Mean p^2^	General-Factor Loading Range
Total sample	5	0.806	0.956	0.842	0.613	0.633	0.449–0.821
Sensitivity sample	4	0.798	0.954	0.837	0.657	0.676	0.484–0.806

***Note*.** Results are based on Schmid–Leiman transformations of exploratory factor solutions fitted to polychoric correlations. ωH = omega hierarchical, the proportion of total-score variance attributable to the general factor; ωT = omega total; ωH/ωT = the proportion of reliable total-score variance attributable to the general factor; ECVG = explained common variance of the general factor; p^2^ = the proportion of each item’s common variance attributable to the general factor. Total sample: N = 562. The sensitivity sample contained one randomly selected respondent from each family (n = 213).

**Table 6:** Standardized item loadings for the FAD-GF and BAFFS.

	FAD-GF				BAFFS			
Items	*λ*	*SE*	*Z*	*p*	*λ*	*SE*	*Z*	*p*
FAD1 ^a^	0.491	0.038	12.88	<0.001				
FAD6	0.782	0.029	26.86	<0.001				
FAD11 ^a^	0.682	0.029	23.89	<0.001				
FAD16	0.687	0.032	21.32	<0.001				
FAD21 ^a^	0.585	0.033	17.52	<0.001				
FAD26	0.633	0.030	20.81	<0.001	0.640	0.035	18.17	<0.001
FAD31 ^a^	0.748	0.024	30.60	<0.001				
FAD36	0.720	0.028	25.70	<0.001				
FAD41 ^a^	0.547	0.039	13.89	<0.001				
FAD46	0.711	0.031	22.58	<0.001				
FAD51 ^a^	0.810	0.026	31.48	<0.001	0.803	0.037	21.73	<0.001
FAD56	0.858	0.020	42.67	<0.001	0.870	0.037	23.42	<0.001
Item pairs	*r* *_res_*	*SE*	*Z*	*p*				
FAD11 ^a^ ~~ FAD21 ^a^	0.563	0.042	13.54	<0.001				
FAD11 ^a^ ~~ FAD26	0.438	0.045	9.68	<0.001				
FAD21 ^a^ ~~ FAD26	0.467	0.038	12.22	<0.001				
FAD16 ~~ FAD36	0.396	0.041	9.62	<0.001				
FAD31 ^a^ ~~ FAD51 ^a^	0.275	0.060	4.59	<0.001				
FAD1 ^a^ ~~ FAD41 ^a^	0.284	0.046	6.15	<0.001				
FAD41 ^a^ ~~ FAD46	0.354	0.050	7.13	<0.001				

***Note*.** *N* = 562 (213 clusters). Values are completely standardized estimates. λ = standardized loading; r_res_ = standardized residual correlation after accounting for the general factor. The FAD-GF estimates are derived from exploratory Model 6, in which the seven residual correlations were estimated simultaneously and all other item residual covariances were fixed to zero. The BAFFS loadings were estimated in a separate cluster-adjusted WLSMV model. Because this three-indicator model was just-identified (df = 0), its global fit indices were necessarily perfect and did not provide a test of unidimensionality. Superscript ^a^ denotes items reverse-scored before analysis.

**Table 7:** Measurement invariance of the 12-item General Functioning (FAD-GF) Scale across respondent roles and diagnostic groups.

**Panel A. Model Fit**									
**Comparison**	**Model**	**χ^2^ (df)**	***p***	**CFI**	**TLI**	**RMSEA [90% CI]**			**SRMR**
Parents vs. adolescents	Configural	170.37 (94)	<0.001	0.987	0.981	0.054 [0.041, 0.067]			0.042
	Threshold	179.89 (106)	<0.001	0.987	0.984	0.050 [0.037, 0.062]			0.042
	Metric	194.73 (117)	<0.001	0.987	0.985	0.049 [0.036, 0.061]			0.044
	Scalar	269.78 (128)	<0.001	0.975	0.975	0.063 [0.052, 0.073]			0.044
	Partial scalar	207.72 (125)	<0.001	0.986	0.985	0.049 [0.037, 0.060]			0.044
	Partial strict	218.64 (137)	<0.001	0.986	0.986	0.046 [0.034, 0.057]			0.047
Fathers vs. mothers	Configural	149.68 (94)	<0.001	0.989	0.984	0.057 [0.039, 0.074]			0.049
	Threshold	165.37 (106)	<0.001	0.988	0.985	0.055 [0.038, 0.071]			0.049
	Metric	169.84 (117)	0.001	0.989	0.988	0.050 [0.032, 0.066]			0.050
	Scalar	172.42 (128)	0.005	0.991	0.991	0.044 [0.025, 0.060]			0.050
	Strict	206.85 (140)	<0.001	0.986	0.987	0.051 [0.036, 0.066]			0.060
Index adolescents vs. siblings	Configural	157.79 (94)	<0.001	0.972	0.960	0.084 [0.060, 0.106]			0.067
	Threshold	165.69 (106)	<0.001	0.974	0.967	0.076 [0.053, 0.098]			0.067
	Metric	170.90 (117)	<0.001	0.976	0.973	0.069 [0.045, 0.090]			0.069
	Scalar	176.82 (128)	0.003	0.978	0.978	0.063 [0.038, 0.084]			0.069
	Strict	187.06 (140)	0.005	0.979	0.980	0.059 [0.034, 0.080]			0.078
ASD vs. AN parents	Configural	141.77 (94)	0.001	0.985	0.979	0.056 [0.036, 0.075]			0.054
	Threshold	153.74 (106)	0.002	0.985	0.981	0.053 [0.033, 0.071]			0.054
	Metric	176.80 (117)	<0.001	0.981	0.979	0.056 [0.039, 0.073]			0.056
	Scalar	205.01 (128)	<0.001	0.976	0.975	0.061 [0.045, 0.076]			0.057
	Strict	258.75 (140)	<0.001	0.963	0.965	0.073 [0.059, 0.086]			0.074
**Panel B. Changes in Fit**									
**Comparison**	**Model comparison**		**Δχ^2^ (Δdf)**		***p***	**ΔCFI**	**ΔTLI**	**ΔRMSEA**	**ΔSRMR**
Parents vs. adolescents	Threshold vs. configural		8.66 (12)		0.732	0.000	0.003	−0.004	0.000
	Metric vs. threshold		20.45 (11)		0.040	−0.001	0.001	−0.001	0.002
	Scalar vs. metric		63.52 (11)		<0.001	−0.011	−0.010	0.014	0.000
	Partial scalar vs. metric		15.83 (8)		0.045	−0.001	0.000	0.000	0.000
	Partial strict vs. partial scalar		20.45 (12)		0.059	0.000	0.001	−0.003	0.003
Fathers vs. mothers	Threshold vs. configural		17.08 (12)		0.147	−0.001	0.001	−0.002	0.000
	Metric vs. threshold		11.92 (11)		0.370	0.001	0.003	−0.006	0.001
	Scalar vs. metric		6.39 (11)		0.846	0.002	0.003	−0.006	0.000
	Strict vs. scalar		32.08 (12)		0.001	−0.005	−0.004	0.008	0.010
Index adolescents vs. siblings	Threshold vs. configural		6.41 (12)		0.894	0.002	0.007	−0.007	0.000
	Metric vs. threshold		13.28 (11)		0.276	0.003	0.006	−0.007	0.002
	Scalar vs. metric		9.88 (11)		0.541	0.002	0.005	−0.006	0.000
	Strict vs. scalar		16.29 (12)		0.178	0.001	0.003	−0.004	0.009
ASD vs. AN parents	Threshold vs. configural		12.43 (12)		0.412	0.000	0.002	−0.003	0.000
	Metric vs. threshold		23.48 (11)		0.015	−0.004	−0.003	0.003	0.002
	Scalar vs. metric		29.70 (11)		0.002	−0.005	−0.004	0.005	0.000
	Strict vs. scalar		49.11 (12)		<0.001	−0.013	−0.010	0.011	0.017

**Note.** All invariance models retained the seven residual covariances specified in Model 6. All models were estimated for ordered categorical indicators using WLSMV. Model chi-square values are scaled, CFI, TLI, and RMSEA are robust estimates, and chi-square difference tests are scaled and shifted. Change statistics refer to the immediately preceding level, except that the parent-adolescent partial scalar model is compared with the metric model. The parent–adolescent partial scalar model allowed the latent-response intercepts of FAD16, FAD36, and FAD56 to vary, and the partial strict model retained these freed latent-response intercepts.

**Table 8:** Internal consistency coefficients for the FAD-GF and BAFFS across subgroups.

Samples	GF						BAFFS					
	Cronbach’s α			McDonald’s ωT			Cronbach’s α			McDonald’s ωT		
	α	LLCI	ULCI	ω	LLCI	ULCI	α	LLCI	ULCI	ω	LLCI	ULCI
Total sample	0.893	0.873	0.910	0.896	0.878	0.912	0.747	0.696	0.790	0.756	0.707	0.797
Parents	0.897	0.870	0.917	0.901	0.876	0.920	0.744	0.678	0.796	0.752	0.690	0.805
Adolescents	0.888	0.855	0.909	0.891	0.862	0.911	0.749	0.665	0.806	0.774	0.705	0.822
Autism	0.907	0.864	0.932	0.909	0.870	0.934	0.785	0.700	0.841	0.796	0.719	0.852
Anorexia	0.882	0.831	0.914	0.888	0.840	0.919	0.687	0.589	0.785	0.693	0.558	0.792
Diabetes	0.876	0.799	0.919	0.886	0.823	0.925	0.716	0.546	0.816	0.740	0.613	0.836

**Note.** α = Cronbach’s alpha; ωT = McDonald’s omega total, estimated from a one-factor minimum-residual solution based on Pearson correlations among the observed item scores; these estimates are distinct from the multidimensional, polychoric-correlation-based Schmid–Leiman ωT estimates reported in Table 5. The reverse-keyed items were recoded before calculating the coefficients. The 95% confidence intervals were obtained from 2000 bootstrap samples with resampling at the family-cluster level. LLCI = Lower Limit of the 95% Confidence Interval; ULCI = Upper Limit of the 95% Confidence Interval. Diagnostic subgroups consist of parental reports only to ensure consistency with multigroup invariance analyses.

**Table 9:** Descriptive statistics and Spearman’s correlations for FAD-GF and BAFFS across respondent-role and diagnostic groups.

Variables	FAD-GF					BAFFS				
	Adolescent	Adult	Autism	Anorexia	Diabetes	Adolescent	Adult	Autism	Anorexia	Diabetes
M	1.96	1.87	1.99	1.83	1.62	1.85	1.69	1.79	1.67	1.47
SD	0.58	0.55	0.62	0.48	0.48	0.68	0.62	0.71	0.54	0.52
Skewness	0.75	0.96	0.85	0.95	0.80	0.98	1.20	1.02	1.31	0.95
Kurtosis	0.36	1.17	0.47	1.78	−0.01	0.64	1.37	0.42	2.36	0.03
BAFFS score	0.87 ***	0.86 ***	0.85 ***	0.86 ***	0.87 ***	-	-	-	-	-
Age	0.09	−0.01	−0.10	0.08	0.12	0.08	−0.03	−0.06	−0.04	0.09
Gender (women)	0.04	−0.05	0.02	−0.00	−0.33 *	0.01	−0.03	0.02	0.05	−0.38 **
Education level		0.03	0.00	−0.04	0.25		0.04	0.00	−0.02	0.21
Number of children		−0.13 *	−0.12	−0.04	−0.08		−0.12 *	−0.09	−0.09	−0.12
Perceived Stress (PSS-4)	0.43 ***	0.42 ***	0.41 ***	0.34 ***	0.44 **	0.37 ***	0.32 ***	0.33 ***	0.26 ***	0.34
Wellbeing (WHO-WBI)	−0.23 **	−0.24 ***	−0.25 **	−0.12	−0.44 **	−0.22 **	−0.22 ***	−0.23 *	−0.11	−0.44 ***
Relationship satisfaction		−0.58 ***	−0.72 ***	−0.43 ***	−0.44 **		−0.55 ***	−0.67 ***	−0.41 ***	−0.54 **
Child-related dyadic coping satisfaction		−0.46 ***	−0.57 ***	−0.32 ***	−0.46 **		−0.39 ***	−0.50 ***	−0.25**	−0.43 **
Child-related dyadic coping efficacy		−0.49 ***	−0.56 ***	−0.36 ***	−0.62 ***		−0.41 ***	−0.48 ***	−0.29 ***	−0.62 ***
Relationship-related dyadic coping satisfaction		−0.55 ***	−0.67 ***	−0.40 ***	−0.40 *		−0.46 ***	−0.63 ***	−0.26**	−0.41 *
Relationship-related dyadic coping efficacy		−0.56 ***	−0.68 ***	−0.41 ***	−0.44 *		−0.48 ***	−0.65 ***	−0.27 ***	−0.44 *
Everyday dyadic coping satisfaction		−0.49 ***	−0.59 ***	−0.39 ***	−0.31		−0.37 ***	−0.46 ***	−0.26 ***	−0.38 *
Everyday dyadic coping efficacy		−0.51 ***	−0.62 ***	−0.38 ***	−0.46 **		−0.40 ***	−0.52 ***	−0.25 ***	−0.47 **

***Note*.** All reported associations are Spearman rank correlations. Statistical significance was evaluated using regressions of standardized ranks with CR2 cluster-robust standard errors, family ID as the clustering variable, and Satterthwaite-adjusted degrees of freedom. The reported *p*-values were not adjusted for multiple comparisons. * *p* < 0.05; ** *p* < 0.01; *** *p* < 0.001. Diagnostic groups (Autism, Anorexia, Diabetes) consist of parental reports only. Data on education level, number of children, relationship satisfaction, and dyadic coping variables were collected exclusively from the parent sample. FAD-GF = General Functioning (12-item); BAFFS = Brief Assessment of Family Functioning Scale (3-item); WHO-WBI = WHO Well-Being Index; PSS-4 = Perceived Stress Scale.

## Data Availability

The data are not publicly available due to ethical, privacy, and data-protection restrictions relating to sensitive clinical and family-level information.

## Supplementary Materials

The following supporting information can be downloaded at: https://www.mdpi.com/article/10.3390/bs16101753/s1.

## References

[B1-behavsci-16-01753] Alaofè H., Yurika R., Okechukwu A., Hounkpatin W. A., Medenou L., Ehiri J. (2026). Translation, cultural adaptation, and psychometric validation of the McMaster family assessment device’s general functioning subscale: A cross-sectional study among African adults with type 2 diabetes. Journal of Diabetes Research.

[B2-behavsci-16-01753] Almeida A. C., Leandro M. E., Pereira M. G. (2020). Psychometric properties of the Portuguese adaptation of general functioning of family assessment device: A comparative study. Families, Systems, & Health.

[B3-behavsci-16-01753] Assaf M., Hallit R., Malaeb D., Sakr F., Dabbous M., Fekih-Romdhane F., Obeid S., Hallit S. (2025). Psychometric properties of the Arabic version of the McMaster family assessment device–General functioning subscale (FAD-GF) among a sample of Lebanese adults. Cogent Psychology.

[B4-behavsci-16-01753] Babar M. G., Bilal S., Yusof Z. Y., Yusof M., Chinna K., Doss J. G., Pau A. (2021). Cross-cultural adaptation of the general functioning scale of the family into the Malay language. International e-Journal of Science, Medicine & Education.

[B5-behavsci-16-01753] Badovinac S. D., Colasanto M., Flora D. B., Andrade B. F. (2024). Validation of a measure of family functioning in a clinic-referred sample of children with disruptive behavior. Journal of Psychopathology and Behavioral Assessment.

[B6-behavsci-16-01753] Bagley C., Bertrand L., Bolitho F., Mallick K. (2001). Discrepant parent-adolescent views on family functioning: Predictors of poorer self-esteem and problems of emotion and behaviour in British and Canadian adolescents. Journal of Comparative Family Studies.

[B7-behavsci-16-01753] Bech P., Gudex C., Johansen Staehr K. (1996). The WHO (Ten) well-being index: Validation in diabetes. Psychotherapy and Psychosomatics.

[B8-behavsci-16-01753] Berge J. M., Wall M., Larson N., Eisenberg M. E., Loth K. A., Neumark-Sztainer D. (2014). The unique and additive associations of family functioning and parenting practices with disordered eating behaviors in diverse adolescents. Journal of Behavioral Medicine.

[B9-behavsci-16-01753] Bihun J. T., Wamboldt M. Z., Gavin L. A., Wamboldt F. S. (2002). Can the family assessment device (FAD) be used with school aged children?. Family Process.

[B10-behavsci-16-01753] Bodenmann G. (2008). Dyadisches coping inventar: Test manual *[Dyadic coping inventory: Test manual]*.

[B11-behavsci-16-01753] Boterhoven de Haan K. L., Hafekost J., Lawrence D., Sawyer M. G., Zubrick S. R. (2015). Reliability and validity of a short version of the general functioning subscale of the McMaster family assessment device. Family Process.

[B12-behavsci-16-01753] Branje S., Laursen B., Collins W. A., Vangelisti A. L. (2013). Parent–child communication during adolescence. The Routledge handbook of family communication.

[B13-behavsci-16-01753] Byles J., Byrne C., Boyle M. H., Offord D. R. (1988). Ontario child health study: Reliability and validity of the general functioning subscale of the McMaster family assessment device. Family Process.

[B14-behavsci-16-01753] Bylund A., Årestedt K., Benzein E., Thorell A., Persson C. (2016). Assessment of family functioning: Evaluation of the general functioning scale in a Swedish bariatric sample. Scandinavian Journal of Caring Sciences.

[B15-behavsci-16-01753] Chen F. F. (2007). Sensitivity of goodness of fit indexes to lack of measurement invariance. Structural Equation Modeling: A Multidisciplinary Journal.

[B16-behavsci-16-01753] Cohen S., Williamson G. M., Spacapan S., Oskamp S. (1988). Perceived stress in a probability sample of the United States. The social psychology of health.

[B17-behavsci-16-01753] Cong C. W., Tan S. A., Nainee S., Tan C.-S. (2022). Psychometric qualities of the McMaster family assessment device–General functioning subscale for Malaysian samples. International Journal of Environmental Research and Public Health.

[B18-behavsci-16-01753] Cooke D., Marais I., Cavanagh R., Kendall G., Priddis L. (2015). Differences between mothers’ and fathers’ ratings of family functioning with the family assessment device: The validity of combined parent scores. Measurement and Evaluation in Counseling and Development.

[B19-behavsci-16-01753] Danis I., Gervai J., Tóth I., Kurimay T., Török S. (2022). Validation and psychometric properties of the family assessment device in Hungary *[Poster Presentation (P11)]*. 11th Conference of the European Family Therapy Association—Systemic Resonances and Interferences, Slovenia.

[B20-behavsci-16-01753] Danis I., Lakatos K., Ney K., Tóth I., Gervai J. (2005). A szülői és a tágabb családi környezet kérdőíves vizsgálatának lehetőségei. Alkalmazott Pszichológia.

[B21-behavsci-16-01753] De Los Reyes A. (2011). Introduction to the special section: More than measurement error: Discovering meaning behind informant discrepancies in clinical assessments of children and adolescents. Journal of Clinical Child & Adolescent Psychology.

[B22-behavsci-16-01753] De Los Reyes A., Kazdin A. E. (2005). Informant discrepancies in the assessment of childhood psychopathology: A critical review, theoretical framework, and recommendations for further study. Psychological Bulletin.

[B23-behavsci-16-01753] De Los Reyes A., Thomas S. A., Goodman K. L., Kundey S. M. A. (2013). Principles underlying the use of multiple informants’ reports. Annual Review of Clinical Psychology.

[B24-behavsci-16-01753] Epstein N. B., Baldwin L. M., Bishop D. S. (1983). The McMaster family assessment device. Journal of Marital and Family Therapy.

[B25-behavsci-16-01753] Epstein N. B., Bishop D. S., Levin S. (1978). The McMaster model of family functioning. Journal of Marital and Family Therapy.

[B26-behavsci-16-01753] Fornell C., Larcker D. F. (1981). Evaluating structural equation models with unobservable variables and measurement error. Journal of Marketing Research.

[B27-behavsci-16-01753] Furr R. M. (2011). Scale construction and psychometrics for social and personality psychology.

[B28-behavsci-16-01753] Georgiades K., Boyle M. H., Jenkins J. M., Sanford M., Lipman E. (2008). A multilevel analysis of whole family functioning using the McMaster family assessment device. Journal of Family Psychology.

[B29-behavsci-16-01753] Hamilton E., Carr A. (2016). Systematic review of self-report family assessment measures. Family Process.

[B30-behavsci-16-01753] Hausken S. E. S., Lie H. C., Lien N., Sleddens E. F. C., Melbye E. L., Bjelland M. (2019). The reliability of the general functioning scale in Norwegian 13–15-year-old adolescents and association with family dinner frequency. Nutrition Journal.

[B31-behavsci-16-01753] Izzo F., Baiocco R., Pistella J. (2022). Children’s and adolescents’ happiness and family functioning: A systematic literature review. International Journal of Environmental Research and Public Health.

[B32-behavsci-16-01753] Jorgensen T. D., Pornprasertmanit S., Schoemann A. M., Rosseel Y. (2026). semTools: Useful tools for structural equation modeling *(R package version 0.5-8)*.

[B33-behavsci-16-01753] Keitner G. I., Fodor J., Ryan C. E., Miller I. W., Bishop D. S., Epstein N. B. (1991). A cross-cultural study of major depression and family functioning. The Canadian Journal of Psychiatry.

[B34-behavsci-16-01753] Keitner G. I., Ryan C. E., Fodor J., Miller I. W., Epstein N. B., Bishop D. S. (1990). A cross-cultural study of family functioning. Contemporary Family Therapy.

[B35-behavsci-16-01753] Körün A. B., Satıcı S. A., Keitner G. I. (2025). Adaptation of general family functioning assessment device (GF-FAD): Its association with family adaptability, family belongingness and mental well-being. Current Psychology.

[B36-behavsci-16-01753] Kurimay T. (1994). Alkoholbetegek és családjainak multigenerációs és multiaxiális jellemzői *[Multigenerational and multi-axial characteristics of people with alcohol use disorders and their families] [Kandidátusi értkezés, Magyar Tudományos Akadémia]*. PhD thesis.

[B37-behavsci-16-01753] Mansfield A. K., Keitner G. I., Dealy J. (2015). The family assessment device: An update. Family Process.

[B38-behavsci-16-01753] Mansfield A. K., Keitner G. I., Sheeran T. (2019). The Brief Assessment of Family Functioning Scale (BAFFS): A three-item version of the general functioning scale of the family assessment device. Psychotherapy Research.

[B39-behavsci-16-01753] Martos T., Sallay V., Nistor M., Józsa P. (2012). Párkapcsolati megküzdés és jóllét: A Páros Megküzdés Kérdőív magyar változata [Dyadic coping and well-being: Hungarian version of the Dyadic Coping Inventory]. Psychiatria Hungarica.

[B40-behavsci-16-01753] Miller I. W., Epstein N. B., Bishop D. S., Keitner G. I. (1985). The McMaster family assessment device: Reliability and validity. Journal of Marital and Family Therapy.

[B41-behavsci-16-01753] Moos R. H., Insel P. M., Humphrey B. (1974). Preliminary manual for family environment scale, work environment scale, group environment scale.

[B42-behavsci-16-01753] Olson D. H., Bell R., Portner J. (1978). Family adaptability and cohesion evaluation scales *[Unpublished manuscript]*.

[B43-behavsci-16-01753] Pires T., Assis S. G. D., Avanci J. Q., Pesce R. P. (2016). Cross-cultural adaptation of the general functioning scale of the family. Revista de Saúde Pública.

[B44-behavsci-16-01753] Pless I. B., Satterwhite B. (1973). A measure of family functioning and its application. Social Science & Medicine.

[B45-behavsci-16-01753] Posit Software, PBC (2025). RStudio: Integrated development environment for R *(Version 2025.05.1) [Computer software]*.

[B46-behavsci-16-01753] R Core Team (2024). R: A language and environment for statistical computing *(Version 4.4.1) [Computer software]*.

[B47-behavsci-16-01753] Sawyer M. G., Sarris A., Baghurst P. A., Cross D. G., Kalucy R. S. (1988). Family assessment device: Reports from mothers, fathers, and adolescents in community and clinic families. Journal of Marital and Family Therapy.

[B48-behavsci-16-01753] Setiana E. S., Tondok M. S. (2024). Analysis of the psychometric properties of the Indonesian version of the McMaster Family Assessment Device General Functioning subscale (FAD GF-12). Jurnal Ilmiah Psikologi Terapan.

[B49-behavsci-16-01753] Shek D. T. L. (2001). The general functioning scale of the family assessment device: Does it work with Chinese adolescents?. Journal of Clinical Psychology.

[B50-behavsci-16-01753] Skinner H. A., Steinhauer P. D., Santa-Barbara J. (1983). The family assessment measure. Canadian Journal of Community Mental Health.

[B51-behavsci-16-01753] Smilkstein G. (1978). The family APGAR: A proposal for a family function test and its use by physicians. The Journal of Family Practice.

[B52-behavsci-16-01753] Spitzer C., Lübke L., Göbel P., Müller S., Krogmann D., Brähler E., Reis O., Lincke L., Kölch M. (2022). Die Erfassung des allgemeinen familiären Funktionsniveaus: Psychometrische evaluation der deutschen version der brief Assessment of family functioning scale [Assessment of general family functioning: Psychometric evaluation of the German version of the brief assessment of family functioning scale]. Psychotherapie, Psychosomatik, Medizinische Psychologie.

[B53-behavsci-16-01753] Staccini L., Tomba E., Grandi S., Keitner G. I. (2014). The evaluation of family functioning by the Family Assessment Device: A systematic review of studies in adult clinical populations. Family Process.

[B54-behavsci-16-01753] Stauder A., Konkolÿ Thege B. (2006). Az Észlelt Stressz Kérdőív (PSS) magyar verziójának jellemzői [Characteristics of the hungarian version of the perceived stress scale (PSS)]. Mentálhigiéné és Pszichoszomatika.

[B55-behavsci-16-01753] Susánszky É., Konkolÿ Thege B., Stauder A., Kopp M. (2006). A WHO Jól-lét Kérdőív rövidített (WBI-5) magyar változatának validálása a Hungarostudy 2002 országos lakossági egészségfelmérés alapján. Mentálhigiéné és Pszichoszomatika.

[B56-behavsci-16-01753] Thomas P. A., Liu H., Umberson D. (2017). Family relationships and well-being. Innovation in Aging.

[B57-behavsci-16-01753] Vandenberg R. J., Lance C. E. (2000). A review and synthesis of the measurement invariance literature: Suggestions, practices, and recommendations for organizational research. Organizational Research Methods.

[B58-behavsci-16-01753] Wu H., Estabrook R. (2016). Identification of confirmatory factor analysis models of different levels of invariance for ordered categorical outcomes. Psychometrika.

[B59-behavsci-16-01753] Yap M. B. H., Pilkington P. D., Ryan S. M., Jorm A. F. (2014). Parental factors associated with depression and anxiety in young people: A systematic review and meta-analysis. Journal of Affective Disorders.

