# Supplementary material for: Validation of the Hungarian FAD-GF and BAFFS in a Multi-Informant Clinical Sample of Families of Adolescents
Source: Behav Sci (Basel). 2026 Sep 25;16(10):1753. doi: 10.3390/bs16101753 (PMC13701522; doi:10.3390/bs16101753)
Supplement: Supplementary file 1 [file behavsci-16-01753-s001.zip › behavsci-4469046-supplementary.pdf]

Supplementary Materials A. Sample composition

Table S1. Complete Cross-Tabulation of Family-Level Respondent Configurations in the Analytic Sample.

Mother questionnaire	Father questionnaire	Index adolescent not provided. 0 sibling questionnaires	Index adolescent not provided. 1 sibling questionnaire	Index adolescent not provided. 2 sibling questionnaires	Index adolescent provided. 0 sibling questionnaires	Index adolescent provided. 1 sibling questionnaire	Index adolescent provided. 2 sibling questionnaires	Total
Not provided	Not provided	—	0	0	1	0	0	1
Not provided	Provided	1	0	0	1	3	0	5
Provided	Not provided	16	2	0	30	5	0	53
Provided	Provided	62	4	2	41	38	7	154
**Total**		**79**	**6**	**2**	**73**	**46**	**7**	**213**

Note. Values represent numbers of families. The analytic sample comprised 213 families contributing 562 questionnaires: 207 from mothers, 159 from fathers, 126 from index adolescents, and 70 from adolescent siblings. “Provided” indicates that a questionnaire from the specified family role was included in the analytic dataset. A questionnaire not being provided does not establish that the respective family member was absent from the household or declined participation following an invitation.

Supplementary Materials B. Supplementary Analyses

Section S1. Item-Level Descriptive Statistics

Table S2 presents the response-category distributions for all 12 FAD-GF items in the total sample and separately for fathers, mothers, index patients, and siblings. Items marked with a superscript *a* were reverse-scored before calculating the reported statistics, ensuring that higher scores consistently represented more problematic family functioning. There was no item-level missingness, and the distributions are therefore based on all 562 respondents.

In the total sample, item means ranged from 1.52 to 2.22 and standard deviations from 0.67 to 0.95. All items had positively skewed distributions, although the magnitude of skewness varied from slight to marked (0.19–1.51). Consistent with this pattern, response categories 1 and 2 together accounted for 61.8%–90.2% of responses across items, whereas category 4 accounted for 2.5%–10.3%. Kurtosis ranged from −0.79 to 1.90.

Table S2. Item-Level Response Distributions and Descriptive Statistics for the FAD-GF in the Total Sample and by Family Role.

Sample	Response category / Descriptive statistics	FAD1[a]	FAD6	FAD11[a]	FAD16	FAD21[a]	FAD26	FAD31[a]	FAD36	FAD41[a]	FAD46	FAD51[a]	FAD56
Total Sample	1	23.5%	61.0%	33.1%	45.4%	23.7%	23.5%	29.0%	35.4%	28.6%	26.3%	56.9%	57.5%
N = 562	2	38.3%	29.2%	39.1%	41.3%	40.7%	47.9%	39.0%	46.6%	45.7%	61.7%	31.5%	32.0%
	3	30.6%	6.4%	21.2%	10.5%	27.9%	23.8%	21.7%	12.5%	20.3%	9.4%	7.5%	7.7%
	4	7.7%	3.4%	6.6%	2.8%	7.7%	4.8%	10.3%	5.5%	5.3%	2.5%	4.1%	2.8%
	M	2.22	1.52	2.01	1.71	2.20	2.10	2.13	1.88	2.02	1.88	1.59	1.56
	SD	0.89	0.76	0.90	0.77	0.89	0.81	0.95	0.83	0.84	0.67	0.80	0.76
	Skewness	0.19	1.51	0.52	0.93	0.27	0.36	0.46	0.81	0.50	0.65	1.36	1.33
	Kurtosis	-0.79	1.90	-0.57	0.48	-0.70	-0.37	-0.72	0.25	-0.33	1.14	1.36	1.36
Father	1	23.9%	62.3%	30.2%	38.4%	20.8%	20.8%	28.3%	26.4%	27.0%	25.2%	56.6%	63.5%
n = 159	2	39.0%	27.7%	43.4%	47.8%	44.0%	53.5%	44.7%	56.6%	47.2%	63.5%	30.2%	27.0%
	3	29.6%	7.5%	22.0%	11.9%	32.1%	25.2%	17.6%	12.6%	23.3%	10.1%	10.1%	8.2%
	4	7.5%	2.5%	4.4%	1.9%	3.1%	0.6%	9.4%	4.4%	2.5%	1.3%	3.1%	1.3%
Mother	1	25.6%	69.1%	36.7%	37.7%	29.0%	28.5%	31.4%	30.9%	34.3%	28.5%	59.4%	62.3%
n = 207	2	40.1%	24.2%	36.7%	49.8%	42.0%	46.4%	34.3%	50.7%	45.4%	62.8%	30.4%	30.0%
	3	26.6%	3.9%	19.3%	9.7%	21.7%	21.3%	23.7%	11.6%	15.9%	6.8%	4.8%	5.3%
	4	7.7%	2.9%	7.2%	2.9%	7.2%	3.9%	10.6%	6.8%	4.3%	1.9%	5.3%	2.4%
Index	1	24.6%	49.2%	31.0%	59.5%	21.4%	19.8%	26.2%	44.4%	28.6%	27.8%	54.8%	44.4%
n = 126	2	33.3%	38.1%	38.9%	27.8%	35.7%	42.9%	38.9%	35.7%	40.5%	54.0%	31.7%	42.1%
	3	33.3%	7.9%	22.2%	9.5%	27.8%	26.2%	22.2%	15.1%	21.4%	12.7%	9.5%	7.9%
	4	8.7%	4.8%	7.9%	3.2%	15.1%	11.1%	12.7%	4.8%	9.5%	5.6%	4.0%	5.6%
Sibling	1	14.3%	55.7%	32.9%	58.6%	18.6%	21.4%	28.6%	52.9%	15.7%	20.0%	54.3%	52.9%
n = 70	2	40.0%	31.4%	37.1%	25.7%	38.6%	48.6%	40.0%	31.4%	52.9%	68.6%	37.1%	31.4%
	3	40.0%	8.6%	22.9%	11.4%	37.1%	24.3%	24.3%	10.0%	24.3%	10.0%	5.7%	12.9%
	4	5.7%	4.3%	7.1%	4.3%	5.7%	5.7%	7.1%	5.7%	7.1%	1.4%	2.9%	2.9%

Note. Response-category values are percentages calculated within the indicated sample or family-role group. *M*, *SD*, skewness, and excess kurtosis are reported for the total sample. Items marked with a superscript *a* were reverse-scored before analysis; consequently, higher values on every item indicate more problematic family functioning. All 562 respondents had complete data for the 12 FAD-GF items. FAD-GF = General Functioning subscale of the Family Assessment Device; Index = index patient.

Section S2. Confirmatory Factor Analyses

This section provides the R code and detailed diagnostic results for the five prespecified cluster-adjusted ordinal CFA models and the exploratory Model 6 summarized in Table 2 of the main text.

Model 1 – Original model

Model 1 specified the original 12-item FAD-GF as a single latent factor.

R code

```
library(lavaan)

fad_items <- c("FADr1", "FAD6", "FADr11", "FAD16", "FADr21", "FAD26", "FADr31", "FAD36", "FADr41", "FAD46", "FADr51", "FAD56")
model_original <- 'GF =~ FADr1 + FAD6 + FADr11 + FAD16 + FADr21 + FAD26 + FADr31 + FAD36 + FADr41 + FAD46 + FADr51 + FAD56'
fit_original <- cfa(model_original, data = FADGFMOD, ordered = fad_items, estimator = "WLSMV", cluster = "FamID", parameterization = "theta", std.lv = TRUE)

# Model fit
c(converged = lavInspect(fit_original, "converged"), post_check = lavInspect(fit_original, "post.check"))
fitMeasures(fit_original, c("chisq.scaled", "df.scaled", "pvalue.scaled", "cfi.robust", "tli.robust", "rmsea.robust", "rmsea.ci.lower.robust", "rmsea.ci.upper.robust", "srmr"))

# Modification indices
mi_original <- modificationIndices(fit_original, standardized = TRUE, sort. = TRUE)
head(mi_original[, c("lhs", "op", "rhs", "mi", "epc", "sepc.all")], 20)
```

Table S3. Modification indices of Model 1.

LHS	OP	RHS	MI	EPC	SEPC.ALL
FADr11	~~	FADr21	82.96	0.63	0.63
FAD16	~~	FAD36	66.00	0.53	0.53
FADr21	~~	FAD26	43.20	0.43	0.43
FADr41	~~	FAD46	39.21	0.40	0.40
FADr1	~~	FADr41	29.51	0.30	0.30
FADr11	~~	FAD26	27.67	0.39	0.39
FADr31	~~	FADr51	27.28	0.43	0.43
FAD16	~~	FADr21	23.11	-0.47	-0.47
FAD6	~~	FAD56	15.63	0.39	0.39
FADr21	~~	FAD56	15.20	-0.47	-0.47
FADr11	~~	FAD46	13.01	-0.34	-0.34
FADr21	~~	FADr31	11.24	-0.29	-0.29
FADr11	~~	FAD16	10.97	-0.33	-0.33
FADr11	~~	FADr41	10.09	-0.28	-0.28
FADr21	~~	FADr51	9.89	-0.34	-0.34
FADr11	~~	FAD56	9.17	-0.35	-0.35
FADr21	~~	FAD36	8.90	-0.28	-0.28
FADr11	~~	FAD36	8.49	-0.28	-0.28
FAD16	~~	FAD26	8.38	-0.26	-0.26
FADr11	~~	FADr31	7.92	-0.27	-0.27

Note. The 20 largest indices are shown; ~~ = residual covariance, MI = modification index, EPC = expected parameter change, and SEPC.ALL = completely standardized EPC.

Model 2 – Method factor for the reverse-coded items

Model 2 retained the general factor and added an orthogonal method factor defined by the six originally negatively worded, reverse-coded items.

R code

```
# Reverse-wording method factor
model_method <- '
  GF =~ FADr1 + FAD6 + FADr11 + FAD16 + FADr21 + FAD26 + FADr31 + FAD36 + FADr41 + FAD46 + FADr51 + FAD56
  METHOD =~ FADr1 + FADr11 + FADr21 + FADr31 + FADr41 + FADr51
  GF ~~ 0*METHOD'

fit_method <- cfa(model_method, data = FADGFMOD, ordered = fad_items, estimator = "WLSMV", cluster = "FamID",
parameterization = "theta", std.lv = TRUE)

# Model fit
c(converged = lavInspect(fit_method, "converged"), post_check = lavInspect(fit_method, "post.check"))

fitMeasures(fit_method, c("chisq.scaled", "df.scaled", "pvalue.scaled", "cfi.robust", "tli.robust", "rmsea.robust",
"rmsea.ci.lower.robust", "rmsea.ci.upper.robust", "srmr"))

# Factor loadings
method_loadings <- standardizedSolution(fit_method)
subset(method_loadings, op == "=~", select = c(lhs, rhs, est.std, se, z, pvalue))

# Modification indices
mi_method <- modificationIndices(fit_method, standardized = TRUE, sort. = TRUE)
head(mi_method[, c("lhs", "op", "rhs", "mi", "epc", "sepc.all")], 20)
```

Table S4. Standardized factor loadings of Model 2.

LHS	RHS	λ	SE	Z	p
GF	FADr1	0.50	0.04	14.20	< .001
GF	FAD6	0.75	0.03	25.97	< .001
GF	FADr11	0.77	0.02	33.22	< .001
GF	FAD16	0.72	0.03	25.91	< .001
GF	FADr21	0.70	0.03	28.66	< .001
GF	FAD26	0.73	0.02	30.71	< .001
GF	FADr31	0.75	0.02	33.53	< .001
GF	FAD36	0.74	0.02	31.58	< .001
GF	FADr41	0.59	0.03	17.57	< .001
GF	FAD46	0.71	0.03	24.89	< .001
GF	FADr51	0.81	0.03	32.89	< .001
GF	FAD56	0.82	0.02	40.37	< .001
METHOD	FADr1	0.17	0.06	2.99	.003
METHOD	FADr11	-0.41	0.05	-7.65	< .001
METHOD	FADr21	-0.45	0.06	-8.14	< .001
METHOD	FADr31	0.25	0.05	5.48	< .001
METHOD	FADr41	0.18	0.06	3.32	.001
METHOD	FADr51	0.22	0.05	4.15	< .001

Note. λ = standardized factor loading; SE = standard error.

Table S5. Modification indices of Model 2.

LHS	OP	RHS	MI	EPC	SEPC.ALL
METHOD	=~	FAD26	109.19	-0.72	-0.49
FAD16	~~	FAD36	65.18	0.53	0.53
FADr21	~~	FAD26	59.46	0.68	0.68
FADr41	~~	FAD46	37.72	0.40	0.40
FADr11	~~	FAD26	35.11	0.58	0.58
FADr1	~~	FADr41	23.43	0.31	0.31
METHOD	=~	FAD46	22.47	0.38	0.27
FAD16	~~	FADr21	20.92	-0.57	-0.57
FAD6	~~	FAD56	15.22	0.39	0.39
METHOD	=~	FAD56	14.84	0.39	0.22
METHOD	=~	FAD16	13.97	0.32	0.22
FADr21	~~	FAD56	13.12	-0.56	-0.56
FADr11	~~	FAD46	11.78	-0.42	-0.42
FADr31	~~	FADr51	10.66	0.45	0.45
FADr11	~~	FAD16	9.78	-0.40	-0.40
FADr11	~~	FADr21	9.41	1.75	1.75
FAD16	~~	FAD26	8.77	-0.27	-0.27
FAD26	~~	FADr31	8.30	-0.29	-0.29
METHOD	=~	FAD36	8.01	0.23	0.16
FADr11	~~	FAD56	7.87	-0.42	-0.42

Note. The 20 largest indices are shown; =~ = factor loading, ~~ = residual covariance, MI = modification index, EPC = expected parameter change, and SEPC.ALL = completely standardized EPC.

Model 3 – Two-factor solution (Positive–negative wording model)

Model 3 specified two correlated factors defined by the positively and negatively worded items.

R code

```
model_wording <- '
  POSITIVE =~ FAD6 + FAD16 + FAD26 + FAD36 + FAD46 + FAD56
  NEGATIVE =~ FADr1 + FADr11 + FADr21 + FADr31 + FADr41 + FADr51'

fit_wording <- cfa(model_wording, data = FADGFMOD, ordered = fad_items, estimator = "WLSMV", cluster = "FamID",
parameterization = "theta", std.lv = TRUE)

# Model fit
c(converged = lavInspect(fit_wording, "converged"), post_check = lavInspect(fit_wording, "post.check"))
fitMeasures(fit_wording, c("chisq.scaled", "df.scaled", "pvalue.scaled", "cfi.robust", "tli.robust", "rmsea.robust",
"rmsea.ci.lower.robust", "rmsea.ci.upper.robust", "srmr"))

# Loadings and factor correlation
wording_solution <- standardizedSolution(fit_wording)
subset(wording_solution, op == "=~", select = c(lhs, rhs, est.std, se, z, pvalue))
subset(wording_solution, op == "~~" & lhs == "POSITIVE" & rhs == "NEGATIVE", select = c(lhs, op, rhs, est.std, se, z, pvalue))

# Modification indices
mi_wording <- modificationIndices(fit_wording, standardized = TRUE, sort. = TRUE)
head(mi_wording[, c("lhs", "op", "rhs", "mi", "epc", "sepc.all")], 20)
```

Table S6. Standardized Factor Loadings for Model 3.

LHS	RHS	λ	SE	Z	p
POSITIVE	FAD6	0.76	0.03	26.21	< .001
POSITIVE	FAD16	0.73	0.03	26.47	< .001
POSITIVE	FAD26	0.74	0.02	30.68	< .001
POSITIVE	FAD36	0.75	0.02	32.24	< .001
POSITIVE	FAD46	0.72	0.03	24.86	< .001
POSITIVE	FAD56	0.83	0.02	41.05	< .001
NEGATIVE	FADr1	0.51	0.04	14.16	< .001
NEGATIVE	FADr11	0.80	0.02	39.33	< .001
NEGATIVE	FADr21	0.73	0.02	32.60	< .001
NEGATIVE	FADr31	0.75	0.02	35.41	< .001
NEGATIVE	FADr41	0.60	0.03	17.67	< .001
NEGATIVE	FADr51	0.82	0.02	34.09	< .001

Note. λ = standardized factor loading; SE = standard error.

Table S7. Modification indices of Model 3.

LHS	OP	RHS	MI	EPC	SEPC.ALL
FADr11	~~	FADr21	73.73	0.65	0.65
FAD16	~~	FAD36	58.85	0.53	0.53
FAD26	~~	FADr21	52.55	0.49	0.49
FAD46	~~	FADr41	44.49	0.43	0.43
NEGATIVE	=~	FAD26	43.65	3.06	2.06
FAD26	~~	FADr11	34.68	0.46	0.46
FADr1	~~	FADr41	27.07	0.29	0.29
FADr31	~~	FADr51	20.78	0.42	0.42
FAD16	~~	FADr21	19.80	-0.45	-0.45
NEGATIVE	=~	FAD16	15.83	-1.84	-1.27
FADr21	~~	FADr31	15.21	-0.35	-0.35
FADr21	~~	FADr51	13.75	-0.43	-0.43
FADr11	~~	FADr41	13.03	-0.33	-0.33
FAD56	~~	FADr21	12.33	-0.44	-0.44
FADr11	~~	FADr31	12.05	-0.35	-0.35
FAD6	~~	FAD56	11.63	0.36	0.36
FAD16	~~	FAD26	11.13	-0.31	-0.31
FAD46	~~	FADr11	10.24	-0.32	-0.32
FAD26	~~	FAD36	8.62	-0.28	-0.28
FAD16	~~	FADr11	8.45	-0.30	-0.30

Note. The 20 largest indices are shown; =~ = factor loading, ~~ = residual covariance, MI = modification index, EPC = expected parameter change, and SEPC.ALL = completely standardized EPC.

Model 4 – The 6+ model

Model 4 tested the one-factor FAD-GF6+, comprising the six positively worded items.

R code

```
# FAD-GF6+ model
gf6plus_items <- c("FAD6", "FAD16", "FAD26", "FAD36", "FAD46", "FAD56")
model_gf6plus <- ' GF6PLUS =~ FAD6 + FAD16 + FAD26 + FAD36 + FAD46 + FAD56'

fit_gf6plus <- cfa(model_gf6plus, data = FADGFMOD, ordered = gf6plus_items, estimator = "WLSMV", cluster = "FamID",
parameterization = "theta", std.lv = TRUE)

# Model fit
c(converged = lavInspect(fit_gf6plus, "converged"),  post_check = lavInspect(fit_gf6plus, "post.check"))
fitMeasures(fit_gf6plus, c("chisq.scaled", "df.scaled", "pvalue.scaled", "cfi.robust", "tli.robust", "rmsea.robust",
"rmsea.ci.lower.robust", "rmsea.ci.upper.robust", "srmr"))

# Factor loadings
gf6plus_loadings <- standardizedSolution(fit_gf6plus)
subset(gf6plus_loadings, op == "=~", select = c(lhs, rhs, est.std, se, z, pvalue))

# Modification indices
mi_gf6plus <- modificationIndices(fit_gf6plus, standardized = TRUE, sort. = TRUE)
head(mi_gf6plus[, c("lhs", "op", "rhs", "mi", "epc", "sepc.all")], 20)
```

Table S8. Standardized Factor Loadings for Model 4.

LHS	RHS	λ	SE	Z	p
GF6PLUS	FAD6	0.76	0.03	23.92	< .001
GF6PLUS	FAD16	0.78	0.03	28.64	< .001
GF6PLUS	FAD26	0.62	0.03	20.28	< .001
GF6PLUS	FAD36	0.78	0.02	32.76	< .001
GF6PLUS	FAD46	0.70	0.03	22.29	< .001
GF6PLUS	FAD56	0.86	0.02	40.24	< .001

Note. λ = standardized factor loading; SE = standard error.

Table S9. Modification indices of Model 4.

LHS	OP	RHS	MI	EPC	SEPC.ALL
FAD16	~~	FAD36	36.15	0.62	0.62
FAD6	~~	FAD56	11.14	0.47	0.47
FAD6	~~	FAD36	8.27	-0.36	-0.36
FAD16	~~	FAD56	6.63	-0.39	-0.39
FAD36	~~	FAD56	5.30	-0.35	-0.35
FAD16	~~	FAD26	4.21	-0.20	-0.20
FAD6	~~	FAD16	3.96	-0.24	-0.24
FAD26	~~	FAD46	2.11	0.12	0.12
FAD36	~~	FAD46	1.71	-0.13	-0.13
FAD26	~~	FAD36	0.82	-0.09	-0.09
FAD16	~~	FAD46	0.80	-0.09	-0.09
FAD26	~~	FAD56	0.67	0.10	0.10
FAD46	~~	FAD56	0.63	0.10	0.10
FAD6	~~	FAD26	0.63	0.08	0.08
FAD6	~~	FAD46	0.06	0.02	0.02

Note. All possible residual-covariance indices are shown; MI = modification index, EPC = expected parameter change, and SEPC.ALL = completely standardized EPC.

Model 5 – Three-factor solution

Model 5 tested the 11-item structure proposed by Cong et al. (2022), comprising correlated negative-functioning, positive-functioning, and emotional-communication factors.

R code

```
# Cong et al. (2022) three-factor model
cong_items <- c("FADr1", "FAD6", "FADr11", "FAD16", "FADr21", "FAD26", "FADr31", "FAD36", "FADr41", "FAD46", "FADr51")

model_cong <- '
  NEGATIVE =~ FADr1 + FADr31 + FADr41 + FADr51
  POSITIVE =~ FAD6 + FAD16 + FAD36 + FAD46
  EMOCOMM  =~ FADr11 + FADr21 + FAD26
'
fit_cong <- cfa(model_cong, data = FADGFMOD, ordered = cong_items, estimator = "WLSMV", cluster = "FamID",
parameterization = "theta", std.lv = TRUE)

# Model fit
c(converged = lavInspect(fit_cong, "converged"), post_check = lavInspect(fit_cong, "post.check"))
fitMeasures(fit_cong, c("chisq.scaled", "df.scaled", "pvalue.scaled", "cfi.robust", "tli.robust", "rmsea.robust",
"rmsea.ci.lower.robust", "rmsea.ci.upper.robust", "srmr"))

# Loadings and factor correlations
cong_solution <- standardizedSolution(fit_cong)
subset(cong_solution, op == "=~", select = c(lhs, rhs, est.std, se, z, pvalue))
subset(cong_solution, op == "~~" & lhs %in% c("NEGATIVE", "POSITIVE", "EMOCOMM") & rhs %in% c("NEGATIVE", "POSITIVE",
"EMOCOMM") & lhs != rhs, select = c(lhs, op, rhs, est.std, se, z, pvalue))

# Modification indices
mi_cong <- modificationIndices(fit_cong, standardized = TRUE, sort. = TRUE)
head(mi_cong[, c("lhs", "op", "rhs", "mi", "epc", "sepc.all")], 20)
```

Table S10. Standardized Factor Loadings and Factor Correlations for Model 5.

LHS	OP	RHS	λ	SE	Z	p
NEGATIVE	=~	FADr1	0.54	0.04	14.65	< .001
NEGATIVE	=~	FADr31	0.80	0.02	37.75	< .001
NEGATIVE	=~	FADr41	0.63	0.03	18.64	< .001
NEGATIVE	=~	FADr51	0.86	0.02	35.15	< .001
POSITIVE	=~	FAD6	0.76	0.03	23.96	< .001
POSITIVE	=~	FAD16	0.75	0.03	28.01	< .001
POSITIVE	=~	FAD36	0.78	0.02	34.33	< .001
POSITIVE	=~	FAD46	0.74	0.03	24.89	< .001
EMOCOMM	=~	FADr11	0.89	0.02	44.74	< .001
EMOCOMM	=~	FADr21	0.81	0.02	38.98	< .001
EMOCOMM	=~	FAD26	0.80	0.02	33.33	< .001
LHS	OP	RHS	r	SE	Z	p
NEGATIVE	~~	POSITIVE	0.90	0.02	44.23	< .001
NEGATIVE	~~	EMOCOMM	0.70	0.04	20.32	< .001
POSITIVE	~~	EMOCOMM	0.74	0.03	26.39	< .001

Note. λ = standardized factor loading; *r* = standardized factor correlation; SE = standard error.

Table S11. Modification indices of Model 5.

LHS	OP	RHS	MI	EPC	SEPC.ALL
FAD16	~~	FAD36	41.26	0.56	0.56
FADr41	~~	FAD46	40.54	0.46	0.46
EMOCOMM	=~	FAD6	38.52	0.79	0.51
FAD6	~~	FADr11	21.85	0.61	0.61
FADr1	~~	FADr41	17.65	0.26	0.26
NEGATIVE	=~	FAD46	17.46	1.35	0.91
EMOCOMM	=~	FAD16	15.09	-0.50	-0.33
FAD16	~~	FADr21	9.95	-0.38	-0.38
FAD6	~~	FAD36	7.98	-0.34	-0.34
FADr31	~~	FADr41	7.80	-0.27	-0.27
POSITIVE	=~	FADr1	5.69	-0.62	-0.53
FAD6	~~	FADr21	5.59	0.27	0.27
FAD36	~~	FAD46	5.45	-0.24	-0.24
FADr41	~~	FADr51	5.12	-0.28	-0.28
POSITIVE	=~	FADr21	4.60	-0.30	-0.18
FADr11	~~	FAD26	4.47	-0.33	-0.33
FADr31	~~	FADr51	3.91	0.29	0.29
NEGATIVE	=~	FAD16	3.90	-0.67	-0.44
FADr31	~~	FAD46	3.81	-0.22	-0.22
NEGATIVE	=~	FADr21	3.71	-0.25	-0.15

Note. The 20 largest indices are shown; =~ = factor loading, ~~ = residual covariance, MI = modification index, EPC = expected parameter change, and SEPC.ALL = completely standardized EPC.

Model 6 – Exploratory one-factor model with local dependencies

Although Model 6 is presented here alongside the CFA models for completeness, it was not a prespecified, theory-driven model. It was developed exploratorily following the dimensionality and general-factor analyses reported in Section S3. The seven residual covariances were selected primarily from the recurring local item patterns identified in those analyses, with the modification-index patterns from Models 1–5 and item-content overlap serving as supporting evidence. No additional residual covariances were introduced after inspection of Model 6.

R code

```
# Model 6: exploratory one-factor model with seven residual covariances representing local dependencies
model_6 <- ' GF =~ FADr1 + FAD6 + FADr11 + FAD16 + FADr21 + FAD26 + FADr31 + FAD36 + FADr41 + FAD46 + FADr51 + FAD56
  FADr11 ~~ FADr21
  FADr11 ~~ FAD26
  FADr21 ~~ FAD26
  FAD16  ~~ FAD36
  FADr31 ~~ FADr51
  FADr1  ~~ FADr41
  FADr41 ~~ FAD46'

fit_model_6 <- cfa(model_6, data = FADGFMOD, ordered = fad_items, estimator = "WLSMV", cluster = "FamID", parameterization = "theta", std.lv = TRUE)
c(converged = lavInspect(fit_model_6, "converged"), post_check = lavInspect(fit_model_6, "post.check"))
fitMeasures( fit_model_6, c("chisq.scaled",  "df.scaled", "pvalue.scaled","cfi.robust", "tli.robust","rmsea.robust", "rmsea.ci.lower.robust", "rmsea.ci.upper.robust", "srmr" ))
model_6_solution <- standardizedSolution(fit_model_6)
model_6_loadings <- subset(model_6_solution, op == "=~", select = c(lhs, rhs, est.std, se, z, pvalue))
```

```
residual_pairs <- c("FADr11~~FADr21","FADr11~~FAD26", "FADr21~~FAD26", "FAD16~~FAD36", "FADr31~~FADr51",
"FADr1~~FADr41", "FADr41~~FAD46")

pair_id <- paste0(  model_6_solution$lhs,  model_6_solution$op,  model_6_solution$rhs)

model_6_residual_correlations <- model_6_solution[  model_6_solution$op == "~~" &    pair_id %in% residual_pairs,  c("lhs",
"rhs", "est.std", "se", "z", "pvalue")]

model_6_loadings
model_6_residual_correlations
```

Section S3 – Exploratory factor analysis

Section S3 reports parallel analyses and exploratory factor analyses of the 12 FAD-GF items in the total sample and, as a sensitivity analysis, in a subsample containing one randomly selected respondent per family. The four- and five-factor solutions were evaluated using pattern loadings, factor correlations, factor congruence across samples and solutions, and global fit indices (CFI, TLI, RMSEA, RMSR, adjusted RMSR, and BIC).

R code

```
library(psych)
library(dplyr)

# Total sample
efa_items <- FADGFMOD[, fad_items]
R_poly <- psych::polychoric(efa_items, correct = 0.5)$rho
set.seed(20260901)
pa_poly <- psych::fa.parallel( efa_items, fa = "fa", fm = "minres", cor = "poly", n.iter = 1000, quant = 0.95, plot = FALSE)
pa_poly$nfact
round(cbind(Observed = pa_poly$fa.values, Simulated = pa_poly$fa.sim, Resampled = pa_poly$fa.simr), 3)

# One randomly selected respondent per family
set.seed(20260902)
FADGFMOD_one <- FADGFMOD %>%
   group_by(FamID) %>%
   slice_sample(n = 1) %>%
   ungroup()

efa_items_one <- FADGFMOD_one[, fad_items]
R_poly_one <- psych::polychoric(efa_items_one, correct = 0.5)$rho
set.seed(20260903)
pa_poly_one <- psych::fa.parallel( efa_items_one, fa = "fa", fm = "minres", cor = "poly", n.iter = 1000, quant = 0.95, plot = FALSE)
pa_poly_one$nfact
round(cbind(Observed = pa_poly_one$fa.values, Simulated = pa_poly_one$fa.sim, Resampled = pa_poly_one$fa.simr), 3)
```

Table S12. Observed, Simulated, and Resampled Factor Eigenvalues From Parallel Analyses in the Total Sample and the One-Respondent-Per-Family Subsample.

Factors	Total Sample (N = 562)			Sensitivity sample (n = 213)		
	Observed	Simulated	Resampled	Observed	Simulated	Resampled
1	6.014	0.437	0.541	6.333	0.620	0.764
2	0.619	0.195	0.242	0.522	0.323	0.410
3	0.298	0.148	0.184	0.315	0.240	0.308
4	0.181	0.107	0.133	0.224	0.170	0.219
5	0.118	0.070	0.086	0.125	0.108	0.137
6	0.019	0.034	0.042	-0.020	0.049	0.062
7	-0.080	0.000	0.000	-0.065	-0.005	-0.006
8	-0.151	-0.034	-0.043	-0.075	-0.059	-0.077
9	-0.185	-0.069	-0.086	-0.164	-0.115	-0.147
10	-0.252	-0.106	-0.133	-0.243	-0.170	-0.219
11	-0.259	-0.147	-0.182	-0.302	-0.232	-0.297
12	-0.308	-0.196	-0.243	-0.317	-0.308	-0.391

Note. Parallel analyses were conducted using polychoric correlations, minimum residual factor extraction, 1,000 iterations, and the 95th percentile of the simulated and resampled eigenvalues as reference values. The full-sample analysis included all respondents without accounting for within-family dependence. For the sensitivity analysis, one respondent was randomly selected from each family, resulting in an independent-respondent subsample of 213 participants. The parallel analyses suggested five factors in the total sample and four factors in the one-respondent-per-family subsample.

Figure S1. Parallel-Analysis Scree Plots in the Total Sample and the One-Respondent-Per-Family Subsample.

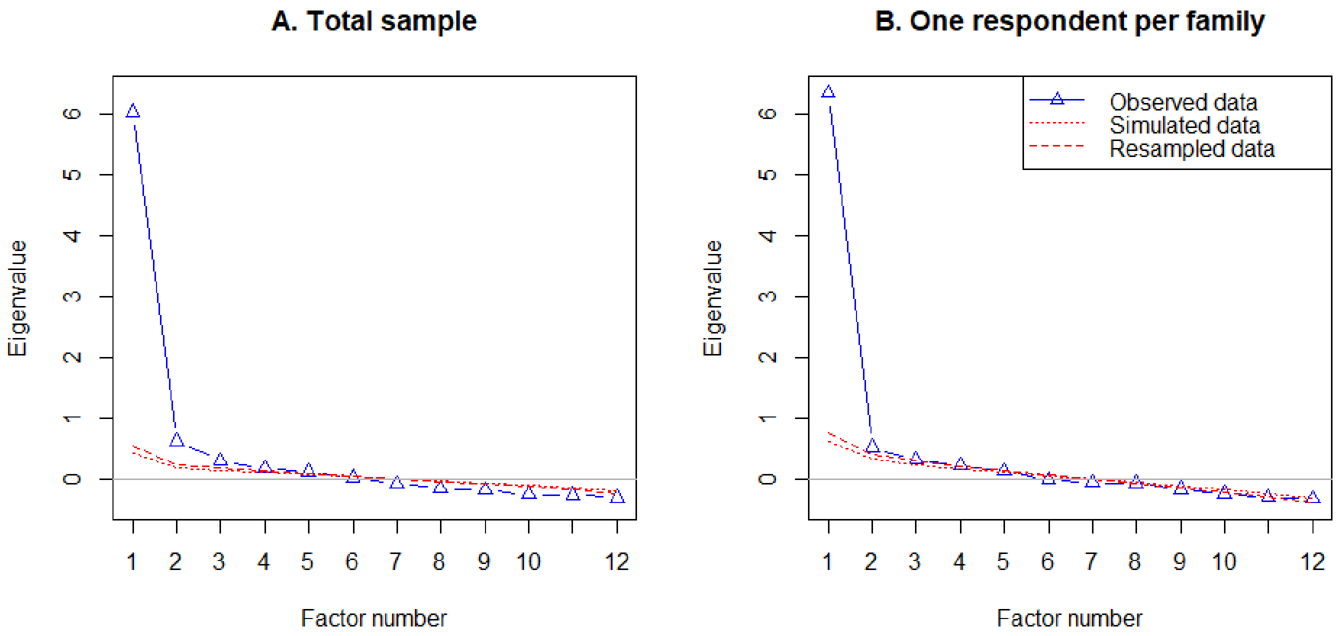

Note. Panel A presents the parallel analysis for the total sample (N = 562), in which multiple respondents from the same family were included and within-family dependence was not modeled. Panel B presents the sensitivity analysis based on one randomly selected respondent from each family (n = 213). Both analyses used polychoric correlations, minimum residual factor extraction, 1,000 iterations, and the 95th percentile of the simulated and resampled eigenvalues as reference values. The analyses suggested retaining five factors in the total sample and four factors in the one-respondent-per-family subsample.

Table S13. Fit and Structural Diagnostics for the Four- and Five-Factor Exploratory Solutions

Panel A. Model-fit indices.

Sample	Factors	Likelihood χ^2 (df)	p	Empirical χ^2 (df)	p	CFI	TLI	RMSEA [90% CI]	RMSR	Adjusted RMSR	BIC
Total sample	4	225.41 (24)	< .001	54.64 (24)	< .001	.951	.865	.122 [.108, .137]	.027	.045	73.45
Total sample	5	111.47 (16)	< .001	20.81 (16)	.186	.977	.904	.103 [.086, .122]	.017	.034	10.17
One respondent per family	4	98.32 (24)	< .001	19.67 (24)	.715	.956	.877	.121 [.097, .146]	.026	.044	−30.35
One respondent per family	5	63.42 (16)	< .001	9.34 (16)	.899	.972	.882	.118 [.089, .150]	.018	.037	−22.36

Panel B. Structural diagnostics

Sample	Factors	Interfactor correlations	Mean item complexity	Matched cross-sample congruence	Boundary or improper estimates
Total sample	4	.44–.62	1.6	.92–.98	None
Total sample	5	.38–.65	1.4	.88–.98	FAD16 and FAD41: h^2 = .995
One respondent per family	4	.40–.64	1.5	.92–.98	FAD41: h^2 = 1.002, u^2 = −.002
One respondent per family	5	.36–.65	1.5	.88–.98	FAD16: h^2 = .996; FAD41: h^2 = .998

Note. The likelihood χ^2, TLI, RMSEA, and BIC are normal-theory indices provided by psych::fa and were treated descriptively because the analyses were based on polychoric correlations. The empirical χ^2 is based on the residual correlation matrix. RMSR = root mean square residual; CFI = Comparative Fit Index; TLI = Tucker–Lewis index; RMSEA = root mean square error of approximation; CI = confidence interval; BIC = Bayesian information criterion; h^2 = communality; u^2 = uniqueness. BIC values are comparable only between solutions fitted to the same sample. Cross-sample congruence values are the matched Tucker coefficients for corresponding factors; because the one-respondent-per-family sample was drawn from the total sample, these coefficients represent sensitivity rather than independent replication.

Table S14. Oblimin-Rotated Pattern Loadings for the Four- and Five-Factor Exploratory Solutions in the Total Sample and the One-Respondent-Per-Family Subsample.

Panel A. Four-factor solution in the total sample

Item	EC	POS/ACC	NEG	DEC	h^2	com
FAD1[a]	—	—	0.35	0.36	0.349	2.39
FAD6	0.39	**0.46**	—	—	0.604	1.97
FAD11[a]	**0.84**	—	—	—	0.779	1.03
FAD16	—	**0.56**	0.30	—	0.597	1.53
FAD21[a]	**0.90**	—	—	—	0.742	1.04
FAD26	**0.69**	—	—	—	0.604	1.11
FAD31[a]	—	—	**0.86**	—	0.807	1.00
FAD36	—	**0.42**	0.36	—	0.581	2.02
FAD41[a]	—	—	—	**0.92**	0.842	1.00
FAD46	—	**0.48**	—	**0.42**	0.628	2.09
FAD51[a]	—	0.26	**0.44**	—	0.667	2.18
FAD56	—	**0.57**	—	—	0.735	1.46

Panel B. Five-factor solution in the total sample

Item	EC	POS	ACC	NEG	DEC	h^2	com
FAD1[a]	—	—	—	0.32	0.32	.318	2.22
FAD6	0.29	**0.56**	—	—	—	.625	1.55
FAD11[a]	**0.81**	—	—	—	—	.771	1.04
FAD16	—	—	**1.02**	—	—	.995†	1.00
FAD21[a]	**0.91**	—	—	—	—	.760	1.03
FAD26	**0.65**	—	—	—	—	.601	1.14
FAD31[a]	—	—	—	**0.90**	—	.862	1.00
FAD36	—	—	**0.44**	0.23	—	.586	1.80
FAD41[a]	—	—	—	—	**0.99**	.995†	1.00
FAD46	—	**0.44**	—	—	0.33	.582	2.14
FAD51[a]	—	0.34	—	**0.43**	—	.686	2.12
FAD56	—	**0.83**	—	—	—	.855	1.03

Panel C. Four-factor solution in the sensitivity sample

Item	EC	POS/ACC	NEG	DEC	h^2	com
FAD1[a]	—	—	0.27	0.37	0.365	2.62
FAD6	0.21	**0.70**	—	—	0.731	1.23
FAD11[a]	**0.97**	—	—	—	0.949	1.00
FAD16	—	**0.47**	0.30	—	0.581	1.85
FAD21[a]	**0.77**	—	—	—	0.694	1.05
FAD26	**0.53**	0.22	—	—	0.513	1.40
FAD31[a]	—	—	**0.88**	—	0.782	1.02
FAD36	—	0.31	0.38	—	0.558	2.28
FAD41[a]	—	—	—	**0.97**	1.002†	1.00
FAD46	—	**0.68**	—	0.29	0.689	1.37
FAD51[a]	0.25	—	**0.69**	—	0.835	1.30
FAD56	—	**0.48**	0.30	—	0.584	1.87

Panel D. Five-factor solution in the sensitivity sample

Item	EC	POS	ACC	NEG	DEC	h^2	com
FAD1[a]	—	—	—	0.31	0.36	.367	2.37
FAD6	0.26	**0.54**	—	—	—	.704	1.91
FAD11[a]	**0.95**	—	—	—	—	.923	1.01
FAD16	—	—	**0.98**	—	—	.996†	1.00
FAD21[a]	**0.82**	—	—	—	—	.719	1.04
FAD26	**0.55**	—	—	—	—	.513	1.25
FAD31[a]	—	—	—	**0.78**	—	.728	1.12
FAD36	—	—	0.33	0.28	—	.568	2.74
FAD41[a]	—	—	—	—	**0.92**	.998†	1.02
FAD46	—	**0.74**	—	—	0.23	.771	1.20
FAD51[a]	—	—	—	**0.80**	—	.893	1.12
FAD56	—	**0.49**	—	0.36	—	.622	2.07

Note. Entries are standardized pattern coefficients from minimum residual exploratory factor analyses with oblimin rotation. Factor columns were reordered across solutions according to their content and Tucker congruence. Absolute loadings below .20 are suppressed, and loadings of $|\lambda| \geq .40$ are shown in bold. EC = emotional communication; POS = positive support and confiding; ACC = acceptance; NEG = negative relational climate; DEC = decision and problem solving; POS/ACC = combined positive-support and acceptance pattern; h^2 = communality; com = item complexity. The factor labels are descriptive and were assigned post hoc. Superscript [a] denotes items reverse-scored before analysis. †Communality was at or above .99; the estimate for FAD41 in Panel C exceeded 1.00 and was accompanied by a small negative uniqueness. Total sample: N = 562. The sensitivity sample contained one randomly selected respondent from each family (n = 213).

Section S4. General-Factor Strength and Schmid–Leiman Analyses

This section reports supplementary unidimensionality indices, complete Schmid–Leiman loading matrices, and reliability decomposition for the factor-defined item subsets in the total sample and the one-respondent-per-family sensitivity sample.

R code

```
# General-factor strength
library(psych)

# Unidimensionality
unidim_total <- psych::unidim(keys = R_poly, n.obs = nrow(efa_items), fm = "minres", check.keys = FALSE,
  nfactors = 5)
unidim_one <- psych::unidim(keys = R_poly_one, n.obs = nrow(efa_items_one), fm = "minres", check.keys = FALSE, nfactors = 4)
print(unidim_total, digits = 3)
print(unidim_one, digits = 3)

# Schmid-Leiman solutions
run_omega <- function(R, n, k) {psych::omega(R, nfactors = k, n.obs = n, fm = "minres", rotate = "oblimin", flip = FALSE, plot =
FALSE, n.rotations = 100)}

set.seed(20260904)
omega_models <- list(Total_5 = run_omega(R_poly, nrow(efa_items), 5), One_per_family_4 = run_omega(R_poly_one,
nrow(efa_items_one), 4))

# Main indices
omega_summary <- function(x) {
  sl <- x$schmid$sl
  specific <- grep("^F[0-9]+\\*$", colnames(sl))
  g_ss <- sum(sl[, "g"]^2)
  group_ss <- colSums(sl[, specific, drop = FALSE]^2)

  c(
    Omega_H = x$omega_h,
    Omega_total = x$omega.tot,
    Omega_H_div_total = x$omega_h / x$omega.tot,
    ECV_general = g_ss / (g_ss + sum(group_ss)),
    Mean_p2 = mean(sl[, "p2"]),
    Min_g_loading = min(sl[, "g"]),
    Max_g_loading = max(sl[, "g"]),
    General_div_largest_group = g_ss / max(group_ss)
  )
}

round(do.call(rbind, lapply(omega_models, omega_summary)), 3)

# Complete Schmid-Leiman results
for (model_name in names(omega_models)) {
  cat("\n", model_name, "\n")
  print(omega_models[[model_name]], digits = 3)
  print(round(omega_models[[model_name]]$schmid$sl, 3))
  print(round(omega_models[[model_name]]$omega.group, 3))
}
```

Table S15. Complete Unidimensionality Diagnostics and First-Factor Dominance.

Sample	Retained factors	u	τ	ρc	α	Average r	Median r	One-factor CFI	ECV_1	F1/F2	MAP
Total sample	5	.927	.949	.976	.921	.493	.486	.809	.720	5.691	.037
Sensitivity sample	4	.943	.962	.981	.929	.522	.531	.797	.784	6.731	.039

Note. The number of factors corresponds to the result of the parallel analysis in each sample. u = unidimensionality index, calculated as the product of τ and ρc; τ = tau-equivalence component; ρc = congeneric-fit component and should not be interpreted as composite reliability; α = standardized coefficient alpha; ECV_1 = explained common variance of the first unrotated factor; F1/F2 = ratio of the first to second factor; MAP = minimum average partial statistic. The four-factor sensitivity solution produced an ultra-Heywood warning involving FAD41; quantities depending on its multifactor extraction should therefore be interpreted cautiously. Total sample: N = 562. The sensitivity sample contained one randomly selected respondent from each family (n = 213).

Table S16. General-Factor Strength Indices for the Parallel-Analysis-Retained Schmid–Leiman Solutions.

Panel A. Score-Level and Common-Variance Indices

Sample	Retained factors	ωH	ωT	ωH/ωT	ECVG	General-factor SS	Largest group-factor SS	SS ratio
Total sample	5	.806	.956	.842	.613	5.291	1.078	4.908
Sensitivity sample	4	.798	.954	.837	.657	5.449	.979	5.565

Panel B. Item-Level Summary Indices

Sample	Retained factors	Mean p^2	SD of p^2	p^2 range	Items with $p^2 > .50$	General-factor loading range
Total sample	5	.633	.169	.304–.845	9/12	.449–.821
Sensitivity sample	4	.676	.129	.405–.844	11/12	.484–.806

Note. Results are based on Schmid–Leiman transformations of exploratory factor solutions fitted to polychoric correlations. The number of retained factors corresponds to the result of the parallel analysis in each sample. ωH = omega hierarchical, the proportion of total-score variance attributable to the general factor; ωT = omega total; ωH/ωT = the proportion of reliable total-score variance attributable to the general factor; ECVG = explained common variance of the general factor; SS = sum of squared loadings; SS ratio = ratio of the general-factor SS to the largest group-factor SS; p^2 = proportion of each item's common variance attributable to the general factor. The count of items with $p^2 > .50$ is descriptive and does not represent a formal cutoff. The four-factor sensitivity solution produced an ultra-Heywood estimate involving FAD41; quantities derived from its specific-factor structure should therefore be interpreted cautiously. Total sample: N = 562. The sensitivity sample contained one randomly selected respondent from each family (n = 213).

Table S17. Item-Level General-Factor Loadings and Proportions of Common Variance Attributable to the General Factor.

Item	Total sample, 5 factors: λg	Total sample, 5 factors: p^2	Sensitivity sample, 4 factors: λg	Sensitivity sample, 4 factors: p^2
FAD1[a]	.449	.633	.484	.644
FAD6	.701	.785	.721	.711
FAD11[a]	.649	.546	.696	.510
FAD16	.701	.494	.682	.800
FAD21[a]	.558	.410	.633	.577
FAD26	.605	.609	.594	.688
FAD31[a]	.742	.639	.738	.697
FAD36	.677	.782	.686	.844
FAD41[a]	.550	.304	.637	.405
FAD46	.664	.758	.675	.661
FAD51[a]	.761	.845	.806	.779
FAD56	.821	.788	.681	.795

Note. λg = standardized loading on the general factor in the Schmid–Leiman solution; p^2 = proportion of the item's common variance attributable to the general factor. The specific group factors were residualized with respect to the general factor through the Schmid–Leiman transformation. Superscript [a] denotes items reverse-scored before analysis. The four-factor sensitivity solution produced an ultra-Heywood estimate involving FAD41; its item-level variance decomposition should therefore be interpreted cautiously. Total sample: N = 562. The sensitivity sample contained one randomly selected respondent from each family (n = 213).

Table S18. Complete Schmid–Leiman Loading Matrices.

Panel A. Total Sample: Five-Group-Factor Solution

Item	g	EC	POS	ACC	DEC	NEG	h^2	u^2	p^2
FAD1[a]	.449	—	—	—	.265	.201	.318	.682	.633
FAD6	.701	.211	.284	—	—	—	.625	.375	.785
FAD11[a]	.649	.588	—	—	—	—	.771	.229	.546
FAD16	.701	—	—	.709	—	—	.995†	.005	.494
FAD21[a]	.558	.666	—	—	—	—	.760	.240	.410
FAD26	.605	.474	—	—	—	—	.601	.399	.609
FAD31[a]	.742	—	—	—	—	.556	.862	.138	.639
FAD36	.677	—	—	.308	—	—	.586	.414	.782
FAD41[a]	.550	—	—	—	.832	—	.995†	.005	.304
FAD46	.664	—	.224	—	.280	—	.582	.418	.758
FAD51[a]	.761	—	—	—	—	.266	.686	.314	.845
FAD56	.821	—	.424	—	—	—	.855	.145	.788

Panel B. Sensitivity sample: Four-Group-Factor Solution

Item	g	EC	POS/ACC	NEG	DEC	h^2	u^2	p^2
FAD1[a]	.484	—	—	—	.296	.365	.635	.644
FAD6	.721	—	.428	—	—	.731	.269	.711
FAD11[a]	.696	.680	—	—	—	.949	.051	.510
FAD16	.682	—	.288	—	—	.581	.419	.800
FAD21[a]	.633	.539	—	—	—	.694	.306	.577
FAD26	.594	.369	—	—	—	.513	.487	.688
FAD31[a]	.738	—	—	.485	—	.782	.218	.697
FAD36	.686	—	—	.207	—	.558	.442	.844
FAD41[a]	.637	—	—	—	.771	1.002†	−.002†	.405
FAD46	.675	—	.413	—	.234	.689	.311	.661
FAD51[a]	.806	—	—	.379	—	.835	.165	.779
FAD56	.681	—	.296	—	—	.584	.416	.795

Note. g = general factor; EC = emotional communication; POS = positive support and confiding; ACC = acceptance; NEG = negative relational climate; DEC = decision and problem solving. The specific-factor labels are descriptive and were assigned post hoc. Absolute specific-factor loadings below .20 are suppressed; general-factor loadings are shown without suppression. h^2 = communality; u^2 = uniqueness; p^2 = proportion of common variance attributable to the general factor. Superscript [a] denotes reverse-scored items. † indicates a boundary or improper communality estimate. Total sample: N = 562. The sensitivity sample contained one randomly selected respondent from each family (n = 213).

Table S19. Reliability Decomposition of Factor-Defined Item Subsets.

Sample	Group factors	Descriptive subset	ωtotal	ωgeneral	ωgroup
Total sample	5	Emotional communication	.877	.459	.417
Total sample	5	Positive support/confiding	.818	.673	.146
Total sample	5	Acceptance	.866	.561	.305
Total sample	5	Decision/problem solving	.799	.474	.325
Total sample	5	Negative relational climate	.857	.660	.197
One respondent per family	4	Emotional communication	.874	.519	.355
One respondent per family	4	Positive support/acceptance	.860	.679	.181
One respondent per family	4	Negative relational climate	.869	.706	.163
One respondent per family	4	Decision/problem solving	.776	.408	.369

Note. The subsets and their labels were derived post hoc from the exploratory group factors. ωtotal represents the reliability of the corresponding subset score; ωgeneral and ωgroup represent the portions attributable to the general factor and the residualized group factor, respectively. The comparatively modest ωgroup values—and the instability or very small size of several factors—do not support interpreting these item subsets as established subscales. Total sample: N = 562. The sensitivity sample contained one randomly selected respondent from each family (n = 213).

Section S5. BAFFS Analyses

This section provides group-specific admissibility diagnostics and ordinal measurement-invariance results for the three-item Brief Assessment of Family Functioning Scale (BAFFS).

Table S20. Group-Specific Loading and Admissibility Diagnostics for the Three-Item BAFFS Model.

Comparison	Group	n	FAD26 λ (residual)	FAD51[a] λ (residual)	FAD56 λ (residual)	Model status
Parents vs. adolescents	Parents	366	.662 (.561)	.877 (.231)	.784 (.386)	Admissible
	Adolescents	196	.572 (.673)	.729 (.469)	1.022 (−.045)†	Improper
Fathers vs. mothers	Fathers	159	.727 (.471)	.801 (.359)	.860 (.260)	Admissible
	Mothers	207	.625 (.609)	.937 (.122)	.730 (.466)	Admissible
Index adolescents vs. siblings	Index adolescents	126	.615 (.622)	.738 (.455)	.975 (.049)	Near-boundary
	Siblings	70	.484 (.766)	.702 (.507)	1.161 (−.349)†	Improper
ASD vs. AN parents	ASD	146	.679 (.538)	.957 (.085)	.796 (.367)	Admissible
	AN	177	.596 (.645)	.756 (.429)	.819 (.329)	Admissible

Note. BAFFS = Brief Assessment of Family Functioning Scale; ASD = autism spectrum disorder; AN = anorexia nervosa. Each group-specific model contained three ordered categorical indicators and was therefore just-identified. Consequently, global model fit could not be evaluated, and the standardized loadings were algebraically determined by the three group-specific polychoric correlations. Values in parentheses are standardized residual variances. The models were estimated using WLSMV with the delta parameterization for diagnostic purposes and without cluster correction; no inferential group comparisons were based on these fits. † The FAD56 loading exceeded 1 and its residual variance was negative, indicating an improper Heywood solution. Because the adolescent and sibling configural models were improper, measurement invariance involving these groups was not interpreted. The index-adolescent solution was technically admissible but close to the boundary.

Table S21. Ordinal Measurement Invariance of the BAFFS Across Parental Roles and Parental Diagnostic Groups.

Panel A. Fathers versus Mothers

**Fit index**	**Configural**	**Threshold**	**Metric**	**Scalar**	**Strict**
χ^2(df)	.00 (0)	6.27 (3)	7.36 (5)	7.50 (7)	17.04 (10)
p	—	.099	.195	.379	.073
CFI	1.000	.995	.996	.999	.988
TLI	1.000	.989	.995	.999	.993
RMSEA [90% CI]	.000 [.000, .000]	.077 [.000, .164]	.051 [.000, .123]	.020 [.000, .095]	.062 [.000, .111]
SRMR	.000	.000	.017	.019	.038
$\Delta\chi^2$(Δdf)	—	6.27 (3)	1.84 (2)	.19 (2)	10.69 (3)
p for $\Delta\chi^2$	—	.099	.399	.910	.014
ΔCFI	—	−.005	.001	.003	−.011
ΔTLI	—	−.011	.006	.004	−.006
ΔRMSEA	—	.077	−.026	−.031	.042
ΔSRMR	—	.000	.017	.002	.019

Panel B. ASD versus AN Parents

**Fit index**	**Configural**	**Threshold**	**Metric**	**Scalar**	**Strict**
χ^2(df)	.00 (0)	2.45 (3)	2.36 (5)	10.67 (7)	11.98 (10)
p	—	.484	.797	.154	.287
CFI	1.000	1.000	1.000	.992	.996
TLI	1.000	1.002	1.007	.993	.997
RMSEA [90% CI]	.000 [.000, .000]	.000 [.000, .123]	.000 [.000, .070]	.057 [.000, .122]	.035 [.000, .096]
SRMR	.000	.000	.005	.026	.026
$\Delta\chi^2$(Δdf)	—	2.45 (3)	.23 (2)	7.81 (2)	.85 (3)
p for $\Delta\chi^2$	—	.484	.890	.020	.837
ΔCFI	—	.000	.000	−.008	.004
ΔTLI	—	.002	.004	−.013	.004
ΔRMSEA	—	.000	.000	.057	−.022
ΔSRMR	—	.000	.005	.021	.001

Note. BAFFS = Brief Assessment of Family Functioning Scale; ASD = autism spectrum disorder; AN = anorexia nervosa. Father–mother models included 159 fathers and 207 mothers; ASD–AN models included 146 ASD and 177 AN parental reports. Models were estimated using WLSMV for ordered categorical indicators. Robust scaled model statistics and scaled-and-shifted chi-square difference tests are reported. Changes are calculated relative to the immediately preceding level. Because the three-item configural models were just-identified, their perfect fit indices were inherent and should not be interpreted as evidence of model fit. Father–mother threshold, metric, and scalar invariance were supported, whereas strict invariance was not. For ASD–AN parents, threshold and metric invariance were supported, but full scalar invariance was not. The strict model is reported for completeness but is not interpreted because scalar invariance was not established. Parent–adolescent and index-adolescent–sibling invariance sequences were not interpreted because their configural solutions included improper FAD56 estimates; see Table S20. The BAFFS invariance models were estimated using WLSMV with the theta parameterization and family ID as the clustering variable.
